# Reprogramming of stroma-derived chemokine networks drives the loss of tissue organization in nodal B cell lymphoma

**DOI:** 10.1038/s43018-026-01136-z

**Published:** 2026-03-25

**Authors:** Felix Czernilofsky, Anna Mathioudaki, Lea Jopp-Saile, Raphael Lutz, Dominik Vonficht, Xi Wang, Christina Schniederjohann, Harald Voehringer, Tobias Roider, Marc-Andrea Baertsch, Claus Rodemer, Henry Löffler-Wirth, Michael Grau, Donnacha Fitzgerald, Johannes Mammen, Jan Kosla, Nora Liebers, Peter-Martin Bruch, Diana Ordoñez-Rueda, Alexander Brobeil, Gunhild Mechtersheimer, Caroline Pabst, Carsten Müller-Tidow, Andreas Trumpp, Marc Seifert, Frank Neumann, Mathias Heikenwälder, Vladimir Benes, Wolfgang Huber, Jörg Distler, Georg Lenz, Hans Binder, Reiner Siebert, Garry P. Nolan, Moritz Gerstung, Judith B. Zaugg, Daniel Hübschmann, Simon Haas, Sascha Dietrich

**Affiliations:** 1 Department of Internal Medicine V, Hematology, Oncology and Rheumatology, University Hospital Heidelberg, Heidelberg, Germany; 2 European Molecular Biology Laboratory (EMBL), Heidelberg, Germany; 3 Molecular Medicine Partnership Unit (MMPU), Heidelberg, Germany; 4 Faculty of Biosciences, University of Heidelberg, Heidelberg, Germany; 5 Division of AI in Oncology, German Cancer Research Centre (DKFZ), Heidelberg, Germany; 6 Heidelberg Institute for Stem Cell Technology and Experimental Medicine (HI-STEM gGmbH), Heidelberg, Germany; 7 Division of Stem Cells and Cancer, German Cancer Research Centre (DKFZ), Heidelberg, Germany; 8 Berlin Institute of Health (BIH), Charité Universitätsmedizin Berlin, Berlin, Germany; 9 Max Delbrück Center for Molecular Medicine in the Helmholtz Association, Berlin Institute for Medical Systems Biology, Berlin, Germany; 10 Department of Hematology, Oncology and Clinical Immunology, Medical Faculty and University Hospital Düsseldorf, Düsseldorf, Germany; 11 Center for Integrated Oncology Aachen Bonn Cologne Düsseldorf (CIO ABCD), Düsseldorf, Germany; 12 Department of Pathology, Stanford University School of Medicine, Stanford, CA USA; 13 Interdisciplinary Centre for Bioinformatics (IZBI), University of Leipzig, Leipzig, Germany; 14 Department of Medicine A, Hematology, Oncology and Pneumology, University Hospital Münster, Münster, Germany; 15 Division of Chronic Inflammation and Cancer, German Cancer Research Center Heidelberg (DKFZ), Heidelberg, Germany; 16 Laboratory of Integrative Biology, Institute of Molecular Genetics of the Czech Academy of Sciences, Prague, Czech Republic; 17 Department of Pathology, University Hospital Heidelberg, Heidelberg, Germany; 18 Kite, a Gilead Company, Santa Monica, CA USA; 19 Department of Rheumatology, Medical Faculty and University Hospital Düsseldorf, Düsseldorf, Germany; 20 Institute of Human Genetics, Ulm University and Ulm University Medical Center, Ulm, Germany; 21 Department of Biomedicine, University of Basel and University Hospital Basel, Basel, Switzerland; 22 Computational Oncology Group (CO), Molecular Precision Oncology Program (MPOP), National Center for Tumor Diseases (NCT) and German Cancer Research Center (DKFZ), Heidelberg, Germany; 23 Innovation and Service Unit for Bioinformatics and Precision Medicine, German Cancer Research Center (DKFZ), Heidelberg, Germany; 24 Division of Translational Precision Medicine, Institute of Human Genetics, University of Heidelberg, Heidelberg, Germany; 25 German Cancer Consortium (DKTK), Heidelberg, Germany; 26 German Cancer Consortium (DKTK), Partner Site Berlin and German Cancer Research Center (DKFZ), Heidelberg, Germany; 27 Precision Healthcare University Research Institute, Queen Mary University of London, London, UK; 28 Cluster of Excellence ImmunoPreCept, Charité Universitätsmedizin Berlin, Berlin, Germany

**Keywords:** Cancer microenvironment, Lymphoid tissues, Cancer

## Abstract

Lymph node (LN) function requires the organization of cells into higher-order spatial units. However, the principles governing LN architecture in health and disease remain poorly understood. Here, we used single-cell and spatial mapping to investigate the mechanisms directing immune cell organization in human LNs and its disruption in architecturally distinct lymphoma entities: indolent follicular lymphoma (FL) and aggressive diffuse large B cell lymphoma (DLBCL). Our data substantiate the central role of LN-resident stromal cells in chemokine-driven lymphocyte zonation and reveal an inflammatory feedback loop fueled by tumor-reactive T cells that triggers stromal remodeling, progressive loss of homeostatic chemokine gradients, and tissue disorganization from a non-malignant state to FL and DLBCL. Loss of homeostatic chemokines was associated with adverse patient survival, identifying the underlying architectural rearrangement as a key event during lymphomagenesis. Collectively, our results highlight the principles of LN organization and suggest how lymphoma-induced microenvironmental reprogramming drives the loss of tissue organization.

## Abstract (web summary)

Czernilofsky et al. identified factors that reprogram stromal cells into an inflammatory, dysfunctional state, leading to the structural disorganization of lymph nodes in B cell lymphoma at single-cell and spatial resolutions.

## Main

The spatial organization of cells within tissues is a fundamental determinant of their physiological functions. Each tissue exhibits a unique architectural layout composed of a three-dimensional arrangement of cells optimized for its specific roles. Perturbations of tissue organization can cause pathophysiological malfunctions that underlie diseases. While single-cell technologies have revolutionized our understanding of cellular composition in tissues, spatially resolved omics can now catalog their spatial localization. However, the principles governing how single cells self-organize into spatially defined microdomains and how disease perturbs this architecture remain poorly understood.

Lymph nodes (LNs) exemplify how spatial architecture determines function. Immune cells that otherwise circulate throughout the body assemble into spatially defined microdomains that fulfill distinct roles in adaptive immunity against pathogens and cancer. A plethora of evidence from mouse models suggests that non-hematopoietic LN stromal cells, comprising both mesenchymal cells (collectively termed fibroblastic reticular cells (FRCs)) and endothelial cells (blood endothelial cells (BECs) and lymphatic endothelial cells (LECs)), have a crucial role in the development and spatial organization of LNs by guiding B and T cell zonation^1,2^. Recent single-cell and spatial analyses have revealed diverse stromal subsets with location-dependent specialization^3–9^: B cell zone FRCs, including follicular dendritic cells (FDCs), guide the follicular organization of B cells by secreting the chemokines C–X–C motif ligand 13 (CXCL13) and CXCL12, as well as the survival factor B cell activating factor (BAFF), while T cell zone FRCs direct T cell migration and homing through the chemokines C–C motif ligand 19 (CCL19) and CCL21 (refs. ^1,2^).

In nodal B cell lymphomas—a heterogeneous group of malignancies varying in genetics, cell of origin, clinical outcome and morphology—the spatial organization of affected LNs is markedly disturbed. Follicular lymphoma (FL) exhibits expansion of follicular B cell zones, whereas diffuse large B cell lymphoma (DLBCL) displays a complete loss of tissue organization. While these distinct growth patterns have long guided lymphoma diagnosis, the molecular and cellular mechanisms driving them remain poorly understood.

Here, we have used single-cell and spatially resolved mapping of human LNs with distinct spatio-organizational patterns to determine the principles of LN tissue organization and its disruption during lymphomagenesis. Our study highlights the role of LN stromal cells in establishing chemokine gradients that orchestrate spatial organization and reveals that disruption of these chemokine networks is a key determinant of the loss of tissue organization in aggressive lymphomas. In particular, an inflammatory feedback loop fueled by tumor-reactive T cells reprograms stromal cells into an inflammatory, dysfunctional state, causing progressive remodeling of the LN ecosystem. Notably, deregulation of LN chemokine networks correlates with adverse clinical outcomes, suggesting that the loss of tissue organization constitutes a crucial event in lymphoma progression.

## Results

### Mapping the spatiocellular principles of lymphoma-induced LN remodeling

**Fig. 1:**
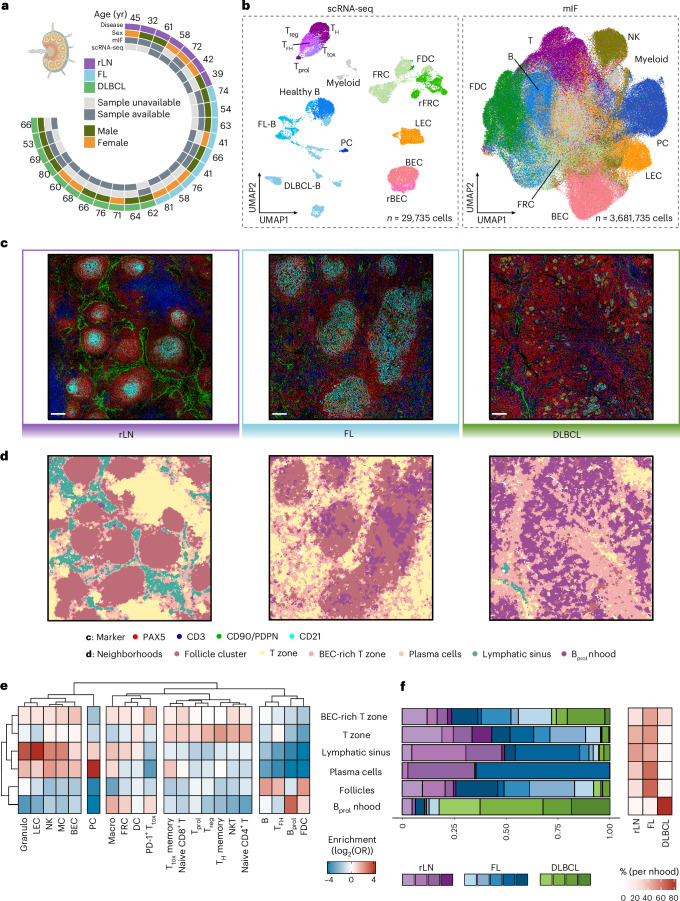
Lymphoma-induced remodeling of the LN cellular ecosystem. **a**, Sample and metadata overview for scRNA-seq and mIF data. **b**, UMAP embeddings of hematopoietic and non-hematopoietic cells obtained using scRNA-seq (left) and mIF (right). **c**, Representative mIF images of rLN (*n* = 4), FL (*n* = 5) and DLBCL (*n* = 4) patient samples. Scale bar, 50 μm. **d**, Segmented mIF images as in **c**, colored by neighborhood identified through *k*-nearest neighbors analysis. **e**, Heatmap depicting the enrichment of subpopulations per spatially defined neighborhood across the complete mIF dataset, based on individual cells pooled across patient samples (*n* = 3,681,735 cells from 13 patient samples). **f**, Left, bar plots illustrating the proportions of identified neighborhoods across patient samples and disease entities. Right, heatmap displaying the percentage of cells per neighborhood and disease entity. UMAP, Uniform Manifold Approximation and Projection; FDC, follicular dendritic cells; rFRC, remodeled FRCs; rBEC, remodeled BECs; PC, plasma cells; NK, natural killer cells; MC, mast cells; DC, dendritic cells; Granulo, granulocytes; Macro, macrophages; T_tox_, cytotoxic T cells; T_prol_, proliferating T cells; T_reg_, T regulatory cells; T_H_, T helper cells; T_FH_, T follicular helper cells; B_prol_, proliferating B cells; nhood, neighborhood; OR, odds ratio. Schematic in **a** created in BioRender; Mathioudaki, A. https://biorender.com/b7kro6a (2025). Source data

**Extended Data Fig. 1:**
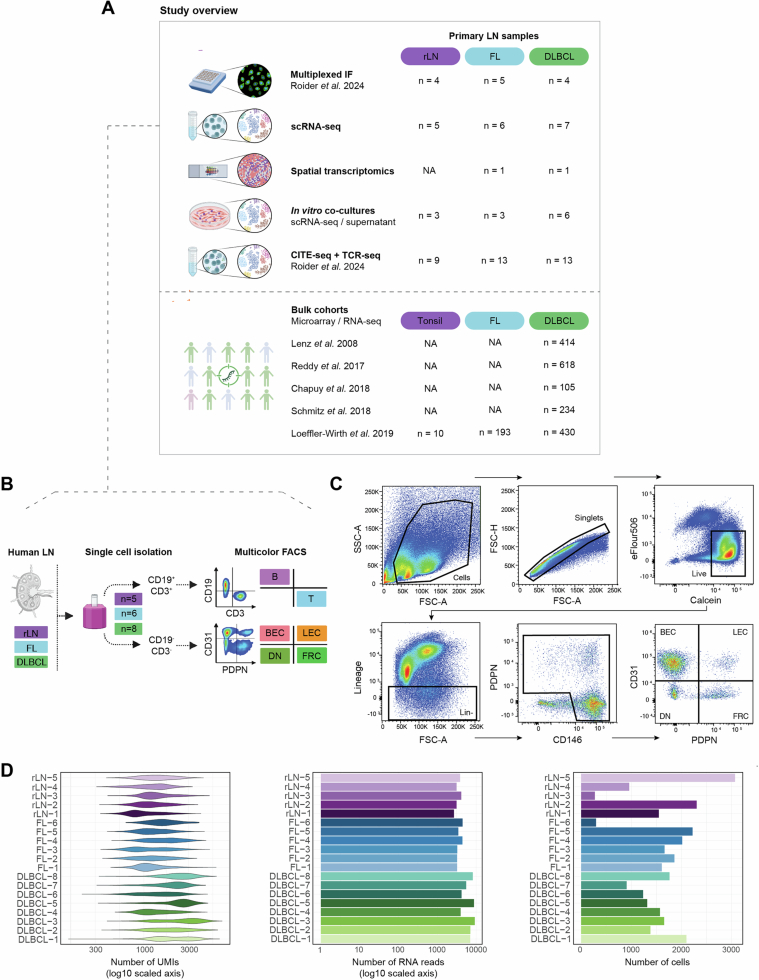
Study overview and scRNA-seq workflow. (**A**) Overview of data layers used in the study. (**B**) Schematic illustrating the strategy for isolation of lymphoid and non-lymphoid populations from primary LNs. (**C**) Sorting strategy for LN-derived non-hematopoietic cells subjected to scRNA-seq analysis. (**D**) Statistics from scRNA-seq output: number of mRNA molecules per cell (left panel), sequencing depth (middle panel), total number of high-quality cells used for downstream analysis (right panel), summarized per sample (rLN n = 5; FL n = 6; DLBCL n = 8 patients). *Abbreviations:* DN = CD31^−^ PDPN^−^ double negative stromal cells, UMIs = unique molecular identifiers. Schematics created in BioRender: **a**, Mathioudaki, A. https://biorender.com/hchbfms (2025); **a**, Mathioudaki, A. https://biorender.com/whkhiun (2025); **b**, Mathioudaki, A. https://biorender.com/vrqn7oj (2025). Source data

To decipher the overarching principles that govern the spatial organization of the human LN and its disruption upon malignant transformation, we performed comprehensive single-cell and spatial analyses of LNs displaying distinct patterns of spatial organization (Fig. 1a,b and Extended Data Fig. 1a). This included non-malignant reactive LNs (rLNs) with a canonical organization into B cell-rich follicles and T cell zones; malignant LNs from patients with FL, characterized by a follicular growth pattern of malignant B cells; and LNs from patients with DLBCL, defined by a highly diffuse growth pattern.

**Extended Data Fig. 2:**
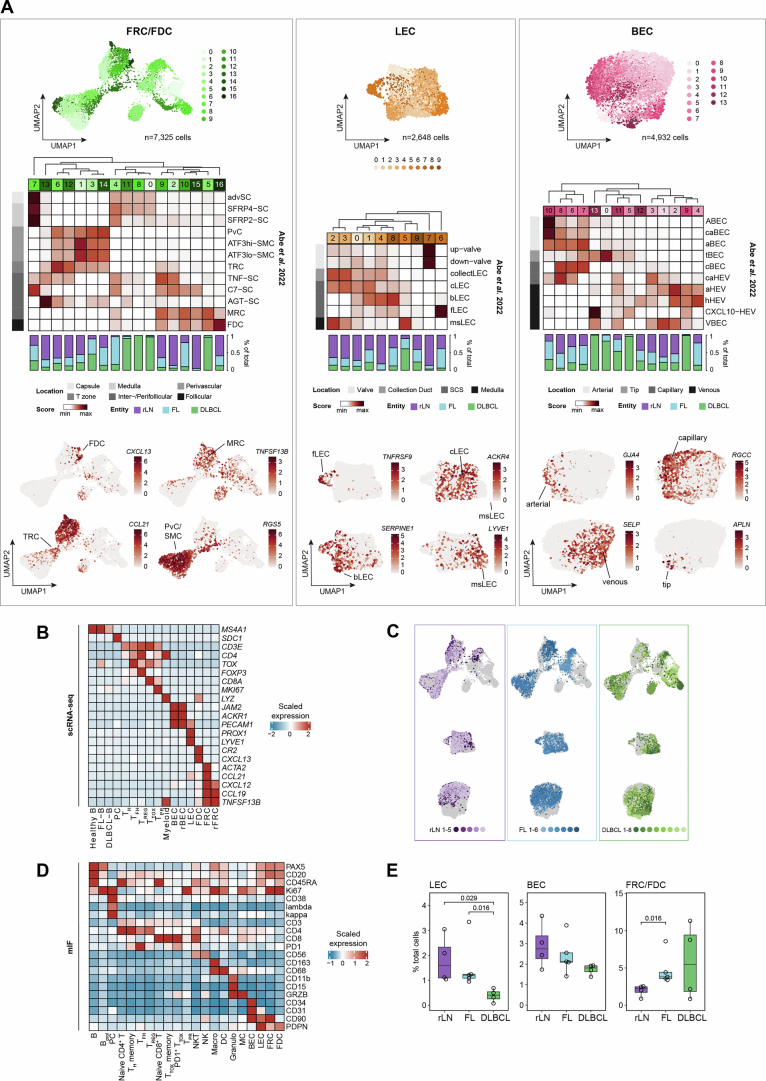
Cluster annotation of stromal cells within scRNA-seq and mIF datasets. (**A**) UMAP representations of scRNA-seq-derived sub-clustered LN stromal cell subsets (top panels). Heatmaps depicting row-scaled scored expression of marker genes per stromal subset identified by a published human LN stromal atlas^9^ as well as contribution of disease entity per cluster (middle panels). UMAP representations showing expression of marker genes for selected stromal subpopulations (bottom panels). (**B**) Heatmap showing scaled average expression of marker genes per identified cell subset. (**C**) UMAP of LN stromal cells as shown in Fig. 1b colored by patient sample. (**D**) Heatmap depicting mIF-derived scaled expression of selected markers across cell types. (**E**) mIF-derived percentages of LECs, BECs and FRCs/FDCs per sample across disease entities. Box plots show the median (center line), interquartile range (bounds), and whiskers extending to 1.5x the interquartile range. Data points represent individual samples (rLN n = 4; FL n = 5; DLBCL n = 4 patients). P-values were calculated using a two-sided unpaired Wilcoxon rank-sum test. *Abbreviations:* FDC = follicular dendritic cells, MRC = marginal reticular cells, TRC = T zone reticular cells, PvC = perivascular cells, SMC = smooth muscle cells, fLEC = subcapsular sinus floor LEC, cLEC = subcapsular sinus ceiling LEC, bLEC = subcapsular sinus bridge LEC, msLEC = medullary sinus LEC, T_PR_ =T_prol_. Source data

To characterize lymphoma-induced changes in LN architecture, we performed single-cell transcriptomics (single-cell RNA sequencing (scRNA-seq)) of primary single cells from rLNs (*n* = 5), FL-LNs (*n* = 6) and DLBCL-LNs (*n* = 8) (Fig. 1a and Supplementary Table 1). Because LNs are dominated by lymphocytes, we experimentally enriched rare mesenchymal and endothelial cells due to their expected key role in spatial LN organization^1,2^ (Extended Data Fig. 1b,c). This yielded 29,735 single cells with strong representation of rare non-hematopoietic cells (Fig. 1b, left, and Extended Data Fig. 1d). Following data integration and unsupervised clustering, we identified 16 cell types and states covering the majority of hematopoietic (B, T and myeloid cells) and non-hematopoietic stromal cell subsets in healthy and lymphomatous LNs^9–12^. The latter included various transcriptionally distinct stromal populations, such as BECs, LECs and FRCs. Among FRCs, we identified B cell zone-derived FRCs characterized by the expression of *TNFSF13B* (BAFF), as well as a subset of FDCs characterized by the expression of *CR2* (CD21) and *CXCL13* (Extended Data Fig. 2a–c and Supplementary Table 2). Stromal clusters in rLNs and FL-LNs mapped to established non-hematopoietic populations. In DLBCL, we observed additional populations of FRCs and BECs that could not be mapped to the rLN and FL-LN populations, suggesting strong stromal remodeling in DLBCL (remodeled FRCs (rFRCs) and remodeled BECs (rBECs); Fig. 1b, left, and Extended Data Fig. 2a–c). To enable comparative analyses across disease entities, we adhered to the broad categories of FRCs, BECs and LECs, unless stated otherwise.

To investigate spatial LN organization, we obtained a 56-plex multiplex immunofluorescence (mIF) dataset comprising 3.68 million cells from rLN (*n* = 4), FL-LN (*n* = 5) and DLBCL-LN (*n* = 4) tissue cores, partially overlapping with our scRNA-seq cohort^11,12^ (Fig. 1a,b, right). We identified 21 hematopoietic and structure-defining non-hematopoietic cell types or states, including PDPN^+^CD31^+^ LECs, PDPN^−^CD31^+^ BECs and PDPN^+^CD90^+^CD31^−^ FRCs. The latter included a distinct subset of B cell zone-residing FDCs defined by CD21 and CXCL13 positivity (Fig. 1b, right, and Extended Data Fig. 2d). To characterize LN spatial organization across lymphoma entities, we defined six spatial neighborhoods using *k*-means clustering (Methods): (1) follicular neighborhoods dominated by B cells, FDCs and T follicular helper (T_FH_) cells (follicles); (2) T cell zones (T zone); (3) T cell zones with high numbers of BECs (BEC-rich T zone); (4) plasma cell-rich neighborhoods (plasma cells); (5) lymphatic sinuses; and (6) neighborhoods characterized by a high number of proliferative B cells (B_prol_ neighborhood) (Fig. 1c,d).

Cellular neighborhoods were largely preserved from rLNs to FL-LNs, yet their structure changed with the malignant expansion of FL cells. As expected, follicular neighborhoods were expanded in both size and number in FL (Fig. 1c–f). In rLNs, FDCs formed compact, round structures at the center of follicles, radially surrounded by B cells, whereas in FL-LNs, FDC networks appeared spatially expanded and poorly demarcated (Fig. 1c,d). Moreover, significantly enlarged plasma cell neighborhoods and an enrichment of BECs in the T cell zone were observed in FL-LNs (Fig. 1c–f). These results suggest FL-driven changes to LN architecture that are specific to stromal cell-defined structures, while overall spatial principles remain intact.

In contrast, the clear separation between B cell- and T cell-dominated neighborhoods was completely disrupted in DLBCL-LNs, consistent with a diffuse growth pattern (Fig. 1c,d). DLBCL-LNs were characterized by a single dominating neighborhood (B_prol_) that mainly harbored proliferating B cells, CD8^+^ T cells, macrophages and expanded FRCs. Remaining FDC-like cells were mostly fragmented into small remnant islands or were entirely absent in some DLBCL-LNs. Moreover, DLBCL-LNs displayed a near-complete depletion of lymphatic vessels and an expansion of non-FDC FRCs, pointing toward a potential role of stromal cell remodeling in the structural reorganization of diffusely growing lymphomas (Fig. 1e,f and Extended Data Fig. 2e).

Together, these analyses unveil global architectural changes in the LN ecosystems of distinct lymphoma entities and provide evidence for the potential involvement of stromal cells in the spatial organization of LNs.

### Chemokine rewiring underlies the loss of LN organization in lymphoma

**Fig. 2:**
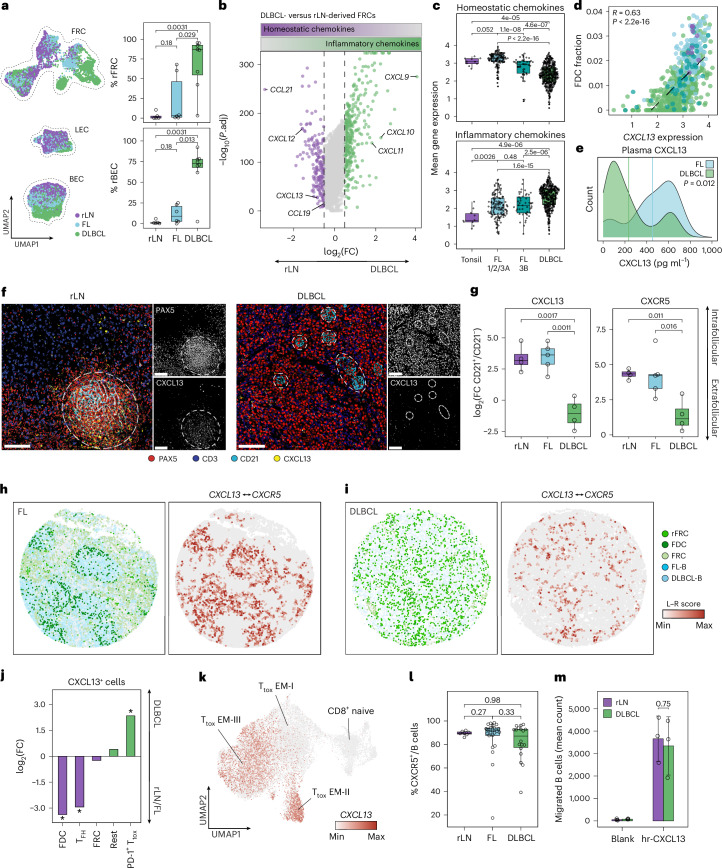
Dysregulation of chemokine networks in FRCs is linked to a diffuse growth pattern in lymphoma. **a**, Left, UMAP of LN stromal cells by entity. Right, percentages of rFRCs and rBECs (rLN *n* = 5, FL *n* = 6, DLBCL *n* = 8 patients). **b**, Differentially expressed genes between rLN-derived (*n* = 2,363 cells) and DLBCL-derived (*n* = 2,983 cells) FRCs (adjusted *P* < 0.05, log(fold change) > 0.5). **c**, Homeostatic (top) and inflammatory (bottom) chemokine expression in bulk data^22^. **d**, Pearson correlation of *CXCL13* expression and CIBERSORTx-derived FDC fractions. **e**, CXCL13 plasma protein levels in FL (*n* = 18 patients) and DLBCL (*n* = 22 patients). Vertical lines indicate the mean per entity. **f**, Exemplary rLN and DLBCL mIF images, representative of *n* = 4 patients per entity. Scale bar, 50 μm. Dashed circles: CD21+ regions. **g**, mIF-derived CXCL13 and CXCR5 signals averaged across four adjacent pairs of CD21^+^ follicular and CD21^−^ extrafollicular regions per sample. **h**,**i**, Spatial transcriptomics plots of FL-LN (**h**) and DLBCL-LN (**i**) cores colored by cell type and *CXCL13*–*CXCR5* ligand–receptor (L–R) score. **j**, mIF-derived enrichment of CXCL13^+^ cells per cell type in DLBCL versus rLN/FL samples. Asterisks indicate *P* < 0.01; exact *P* values are provided in the source data. **k**, *CXCL13* expression in CD8^+^ T cells (*n* = 21,268 cells)^12^. **l**, Percentage of CXCR5^+^ cells within CD3^−^ fractions measured by flow cytometry (rLN *n* = 7, FL *n* = 24, DLBCL *n* = 18 patients). **m**, Migrated rLN- and DLBCL-derived B cells in the Transwell assay (mean ± s.d., *n* = 3 patients per condition). For **c** and **d**: tonsil *n* = 10, FL 1/2/3A *n* = 145, FL 3B *n* = 48, DLBCL *n* = 430 patients. For **f**, **g** and **j**: rLN *n* = 4, FL *n* = 5, DLBCL *n* = 4 patients. *P* values in **a**, **c**, **e**, **g** and **l**: two-sided Wilcoxon rank-sum test. *P* value in **m**: two-sided unpaired Welch’s *t* test. *P* values in **j**: two-sided Fisher’s exact test. *P* values in **c**, **g** and **j** were adjusted using the Benjamini–Hochberg method. Box plots: center line, median; box, interquartile range; whiskers, 1.5× the interquartile range; points, data values. FC, fold change; T_tox_ EM, effector memory cytotoxic T cells; hr, human recombinant. Source data

**Extended Data Fig. 3:**
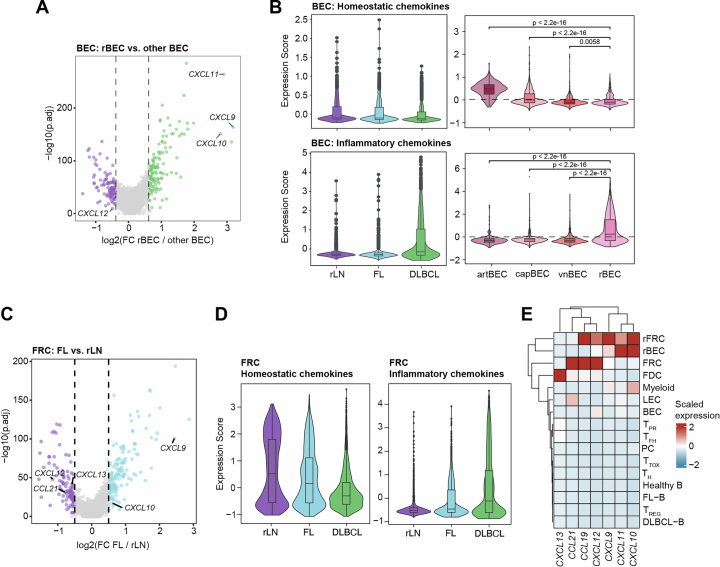
Cross-entity changes in chemokine expression in BECs and FRCs. (**A**) Volcano plot showing differentially expressed genes between rBECs (n = 1,276 cells) and all other BEC subsets (n = 3,656 cells) (Benjamini-Hochberg adjusted p-value < 0.05, logFC > 0.5). (**B**) Scored expression of homeostatic chemokines as well as inflammatory chemokines in all BECs across disease entities (left panels; rLN n = 1,549; FL n = 1,495; DLBCL n = 1,888 cells) as well as across BEC subsets (right panels; artBEC n = 265; capBEC n = 1,981; vnBEC n = 1,410; rBEC n = 1,276 cells). Boxes indicate the median (center line), interquartile range (bounds) and 1.5x interquartile range (whiskers). Data points represent single cells. Dashed line indicates the mean across BEC subsets. P-values were calculated using two-sided unpaired Wilcoxon rank-sum test and adjusted according to Benjamini-Hochberg. (**C**) Volcano plot showing significantly differentially expressed genes between rLN- (n = 2,363 cells) vs. FL-derived (n = 1,979 cells) FRCs/FDCs (Benjamini-Hochberg adjusted p-value < 0.05, logFC > 0.5). (**D**) Scored expression of homeostatic chemokines as well as inflammatory chemokines in FRCs/FDCs across disease entities (rLN n = 2,363; FL n = 1,979; DLBCL n = 2,983 cells). Boxes indicate the median (center line), interquartile range (bounds) and 1.5x interquartile range (whiskers). Data points represent single cells. (**E**) Scaled chemokine expression across cell populations identified by scRNA-seq. *Abbreviations:* artBEC = arterial BEC, capBEC = capillary BEC, vnBEC = venous BEC, rBEC = remodeled BEC, T_PR_ =T_prol_. Source data

Next, we aimed to identify molecular programs that may underlie the loss of tissue architecture in B cell lymphoma. Given the emergence of remodeled stromal cells (rFRCs and rBECs) in DLBCL (Figs. 1b and 2a), we performed differential expression analysis within each stromal compartment in our scRNA-seq data. Specifically, DLBCL- and FL-LNs were each compared separately to rLN samples (Supplementary Table 3). Notably, DLBCL-derived FRCs displayed downregulation of homeostatic chemokines (*CXCL13*, *CXCL12*, *CCL19* and *CCL21*), which act as key players in the development and organization of LN zonation in mice^13–21^ (Fig. 2b). In contrast, interferon (IFN)-inducible inflammatory chemokines (*CXCL9*, *CXCL10* and *CXCL11*) were upregulated in DLBCL-derived FRCs and BECs (Fig. 2b and Extended Data Fig. 3a,b), suggesting lymphoma-induced reprogramming of the chemokine milieu across stromal subsets. In FL-LNs, FRCs showed a similar shift in chemokine expression compared to that in rLNs, although to a lesser extent than in DLBCL-LNs, indicating a gradual change in the LN microenvironment from indolent to more aggressive disease (Extended Data Fig. 3c–e).

**Extended Data Fig. 4:**
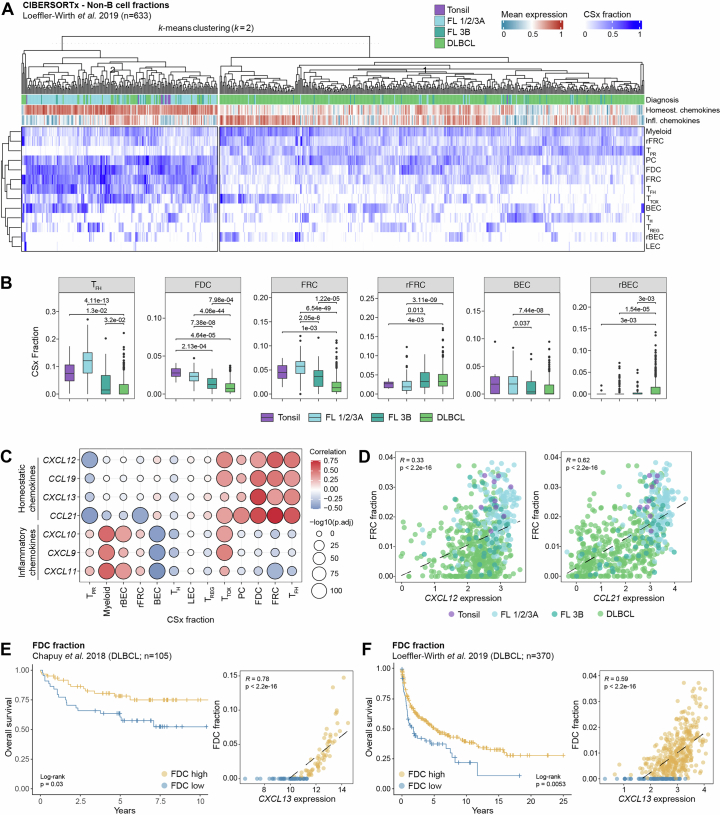
Single-cell-informed cell type deconvolution of bulk transcriptomics data. (**A**) Heatmap showing estimated cell type fractions as determined using CIBERSORTx (CSx) within a large bulk transcriptomics dataset^22^. (**B**) Box plots depicting CSx fractions of exemplary cell types across disease entities. Boxes indicate the median (center line), interquartile range (bounds) and 1.5x interquartile range (whiskers). Data points represent patient samples. P-values were calculated using two-sided unpaired Welch’s t-test and adjusted according to Benjamini-Hochberg. (**C**) Dot plot showing Spearman correlation coefficients of chemokine expression and cell type fractions. Dot sizes represent -log10 FDR-adjusted p-value of correlation tests. (**D**) Scatter plots color-coded by disease entity correlating *CXCL12* (left panel) and *CCL21* (right panel) expression with CSx-derived FRC factions. P-values were calculated using Pearson correlation. (**E**, **F**) Bulk RNA-seq datasets^22,30^ stratified by FDC abundance into high and low samples using maximally selected rank statistics based on CSx fractions, represented in a Kaplan-Meier curve showing overall survival (left panels; log-rank test) and scatter plot of FDC fractions and *CXCL13* expression (right panels; Pearson correlation). T_PR_ =T_prol_. For panels **A**–**D**: tonsil n = 10; FL 1/2/3 A n = 145; FL 3B n = 48; DLBCL n = 430 patients. For panel **E**: FDC-high n = 61; FDC-low n = 44 patients. For panel **F**: FDC-high n = 302; FDC-low n = 68 patients. Source data

LN stromal cells represent only a minor fraction of all cells within LNs, yet they act as the main producers of homeostatic chemokines^1,2^. Consistent with this, the analysis of a large bulk validation cohort (healthy controls *n* = 10; FL 1/2/3A *n* = 145; FL 3B *n* = 48; DLBCL *n* = 430)^22^ confirmed that reprogramming of stromal cells in DLBCL translated into a reduction in the overall synthesis of homeostatic chemokines within the LN microenvironment, along with an upregulation of inflammatory chemokines with a gradual increase from non-malignant controls to FL- and DLBCL-LNs (Fig. 2c). Accordingly, we observed a progressive depletion of FDCs and FRCs, as well as an expansion of rFRCs and rBECs from FL to DLBCL (Extended Data Fig. 4a,b). In line with this, estimates of cell type abundance highlighted a strong correlation between FDC/FRC frequencies and homeostatic chemokine expression, whereas inflammatory chemokine expression was associated with high frequencies of rFRCs and rBECs, linking chemokine levels to stromal cell abundance (Fig. 2d and Extended Data Fig. 4c,d).

**Extended Data Fig. 5:**
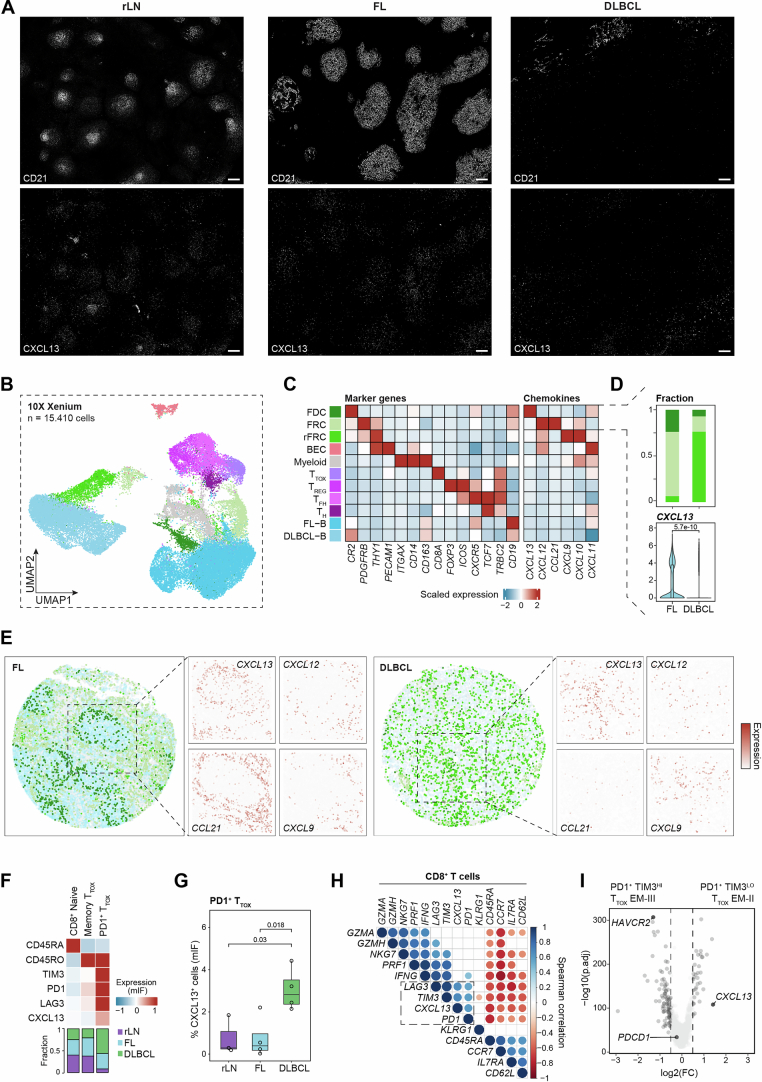
Spatial chemokine profiling identifies CD8^+^ effector memory T cells as an extrafollicular source of CXCL13 in DLBCL. (**A**) Representative triangle-thresholded mIF images showing CD21 (top panels) as well as CXCL13 (bottom panels) signal in rLN (n = 4), FL (n = 5) and DLBCL (n = 4) patient samples. Scale bar indicates 50μm. (**B**) UMAP representation of spatial transcriptomics data colored by cell type (color code depicted in panel **C**). (**C**) Heatmap showing scaled expression of key marker gene expression as well chemokines. (**D**) Stacked bar plot of FDC/FRC/rFRC fractions (top panel) and violin plot of *CXCL13* expression in these subsets (bottom panel). (**E**) Spatial transcriptomics plots of FL (left panels) and DLBCL (right panels) tissue cores colored by cell type (left panels) alongside magnified views of chemokine expression. (**F**) Heatmap showing scaled expression of key T cell markers across CD8^+^ T cell populations as measured using mIF. (**G**) Box plot showing per-sample percentage of CXCL13^+^ cells (as determined using Otsu thresholding) among PD1^+^ CD8^+^ effector memory T cells in the mIF dataset. Boxes indicate the median (center line), interquartile range (bounds) and 1.5x interquartile range (whiskers). Data points represent patient samples. P-values were calculated using a two-sided unpaired Welch’s t-test. (**H**) Spearman correlation coefficients of key T cell markers across all CD8^+^ T cell populations^12^ (n = 21,268 cells). (**I**) Volcano plot showing differentially expressed genes comparing PD1^+^ CD8^+^ effector memory T cell populations^12^ (T_TOX_ EM-II n = 2,018; T_TOX_ EM-III n = 9,208 cells). Labels indicate cluster-identifying genes as well as *CXCL13*. *Abbreviations:* T_TOX_ EM = effector memory cytotoxic T cells. For panels **A**, **F**, **G**: rLN n = 4; FL n = 5; DLBCL n = 4 patients. For panels **B**–**E**: FL n = 1; DLBCL n = 1 patient. Source data

The chemotactic recruitment of CXCR5^+^ B cells to CXCL13^+^ FDCs is a key mechanism in the organization of LN follicles in mice^14,15,17^. In DLBCL, local downregulation of *CXCL13* translated into a reduction in circulating CXCL13 plasma protein levels (Fig. 2e). To investigate whether downregulation of CXCL13 in stromal cells contributes to the loss of tissue organization in DLBCL-LNs, we quantified the spatial expression patterns of CXCL13 at the transcript level (10x Xenium; gene panel in Supplementary Table 4) and at the protein level (mIF). In rLN, the primary sources of CXCL13 were follicle-resident T_FH_ cells and FDCs, establishing orthotopic chemokine gradients centered toward the follicles (Fig. 2f and Extended Data Fig. 5a). Consistent with effective chemoattraction, CXCR5^+^ B cells colocalized with CXCL13^+^ stromal cells (Fig. 2f–i). In contrast, remnant FDC-like islands in DLBCL-LNs were deprived of CXCL13 expression and lost their capacity to recruit CXCR5-presenting B cells, as demonstrated by comparing intrafollicular and extrafollicular signal intensities (Fig. 2g and Extended Data Fig. 5a–e). Both CXCL13 transcript and protein levels, as well as CXCL13–CXCR5 interactions, were diffusely distributed across DLBCL tissue cores with a shift toward extrafollicular regions, indicating a loss of physiologic CXCL13 chemokine gradients (Fig. 2f–i and Extended Data Fig. 5a–e). A global analysis within our dataset and an external single-cell dataset^12^ revealed that, in addition to the loss of CXCL13 in FDC-like cells, *CXCL13* expression was acquired by a newly emerging subpopulation of effector memory PD-1^+^TIM3^lo^CD8^+^ T cells in DLBCL (named T_tox_ EM-II by Roider et al.^12^), further contributing to the breakdown of structure-regulating chemokine gradients (Fig. 2j,k and Extended Data Fig. 5f–i). Importantly, DLBCL B cells maintained their CXCR5 surface expression levels, as measured by flow cytometry, and their capacity to migrate along CXCL13 gradients in transwell assays of primary DLBCL cells (Methods and Fig. 2l,m). This supports a model in which dysregulation of chemokine gradients in the microenvironment—not a direct aberration in malignant B cells—underlies the loss of tissue organization.

Together, these data reveal lymphoma-induced microenvironmental remodeling, including stromal cell reprogramming and the emergence of ectopic chemokine sources, which disrupt chemokine gradients and LN compartmentalization in DLBCL.

### An inflammatory feedback loop drives stromal remodeling in lymphoma

**Fig. 3:**
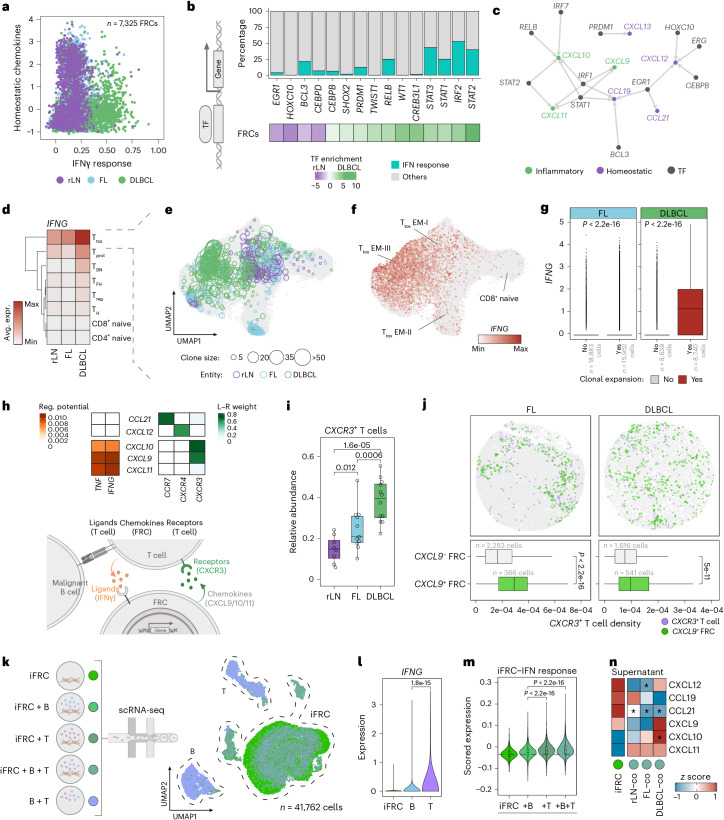
A reprogramming of LN stromal cells from a functional tissue-organizing state to an inflammatory state underlies the loss of tissue organization in lymphoma. **a**, Homeostatic chemokine expression versus the Hallmark IFNγ response score in FRCs (scRNA-seq). **b**, Top 15 differentially active transcription factors in FRCs (bottom) (*n* = 2,363 rLN FRCs; *n* = 2,983 DLBCL FRCs) and fraction of IFNγ response target genes (top). **c**, FRC gene regulatory network of transcription factors targeting homeostatic and inflammatory chemokines. **d**, Average expression of *IFNG* across T cell subsets^12^. **e**,**f**, UMAPs of CD8^+^ T cells from 5′ scRNA-seq data^12^, indicating clonotype size (**e**) and *IFNG* expression (**f**) (*n* = 75,054 cells). **g**, *IFNG* expression in FL and DLBCL-derived CD8^+^ T_tox_ cells colored by clonality status. **h**, T cell-to-FRC interaction analysis. Top, regulatory potential of T cell ligands to FRC target genes (chemokines; left) and ligand–receptor weight (right) (*n* = 2,983 FRCs and *n* = 1,738 T cells in DLBCL; *n* = 2,363 FRCs and *n* = 1,222 T cells in rLN). Bottom, schematic illustrating the proposed model. **i**, Fraction of *CXCR3*^+^ T cells per sample^12^. **j**, Top, spatial transcriptomics plots of FL- and DLBCL-LNs. Bottom, density of *CXCR3*^+^ T cells around *CXCL9*^+^ or *CXCL9*^−^ FRCs. **k**, Schematic (left) and UMAP (right) of scRNA-seq of monocultured and cocultured iFRCs with and without DLBCL-derived B/T cells (*n* = 3 patients). **l**, *IFNG* expression across cell types. **m**, IFNγ response score in monocultured versus cocultured iFRCs. The dashed line indicates the median of monocultures. **n**, *z*-scored chemokine levels in iFRC monoculture versus coculture supernatants with LN-derived B/T cells (rLN, FL and DLBCL; *n* = 3 patients each). Asterisks indicate *P* < 0.05 after a two-sided Welch’s *t* test comparing each condition to monocultures (CXCL12: FL *P* = 0.018; CCL21: rLN *P* = 0.028, FL *P* = 0.003, DLBCL *P* = 0.039; CXCL10: DLBCL *P* = 0.021). For panels **d** and **i**: rLN *n* = 8, FL *n* = 11 and DLBCL *n* = 12 patients. For panels **e**–**g**: rLN *n* = 3, FL *n* = 5 and DLBCL *n* = 3 patient samples. For panels **k**–**m** (iFRC counts per culture condition): iFRC *n* = 8,606; iFRC + B *n* = 10,051; iFRC + T *n* = 6,561; iFRC + B + T *n* = 8,584. *P* values in **g**, **i**, **j**, **l** and **m**: two-sided Wilcoxon rank-sum test. *P* values in **i** and **m** were adjusted using the Benjamini–Hochberg method. Box plots: center line, median; box, interquartile range; whiskers, 1.5× the interquartile range; points, data values. T_DN_, double-negative T cells; TF, transcription factor; L–R, ligand–receptor. Schematic in **h** created in BioRender; Mathioudaki, A. https://biorender.com/t61r11n (2025). Source data

**Extended Data Fig. 6:**
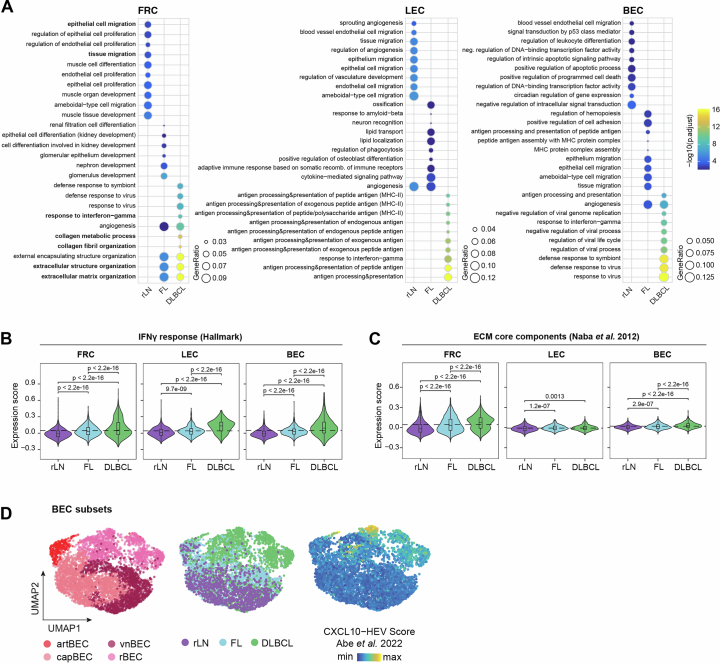
Global inflammatory remodeling across stromal subsets in B cell lymphoma. (**A**) Dot plots of top 10 enriched gene ontology (GO) biological processes terms (sorted by adjusted p-value) for genes significantly upregulated in each disease entity (rLN, DLBCL, FL) and stromal subset (FRC, LEC, BEC). Dot size represents the fraction of enriched genes per term (GeneRatio) and color intensity indicates the -log10 adjusted p-values. Full list provided in Supplementary Table 7. (**B**, **C**) Violin plots depicting single-cell-level expression scores of Hallmark IFNγ response genes (B) and ECM core components^60^ (C) across stromal subsets and disease entities. Boxes indicate the median (center line), interquartile range (bounds) and 1.5x interquartile range (whiskers). Data points represent individual cells (FRC: rLN n = 2,363, FL n = 1,979, DLBCL n = 2,983; LEC: rLN n = 1,485, FL n = 699, DLBCL n = 464; BEC: rLN n = 1,549, FL n = 1495, DLBCL n = 1,888). Dashed line indicates the mean across disease entities. P-values were calculated using the Wilcoxon rank-sum test and adjusted according to Benjamini-Hochberg. (**D**) UMAP representations of BECs colored by subset (left), disease entity (middle) and scored expression of marker genes for *CXCL10*^*+*^ high endothelial venules (CXCL10-HEVs) as described by Abe et al.^9^ (right). Source data

To characterize the pathways driving stromal reprogramming in DLBCL-LNs, we performed Gene Ontology enrichment analysis of differentially expressed genes in stromal cells from non-malignant rLNs and malignant DLBCL-LNs. While rLN stromal cells were enriched for gene programs consistent with normal LN homeostasis, DLBCL stromal cells showed transcriptional reprogramming (Extended Data Fig. 6a). This included increased inflammatory- and IFNγ-driven programs, together with upregulated extracellular matrix genes across all LN stromal populations (Extended Data Fig. 6a–c and Supplementary Tables 5 and 6). Accordingly, FRCs transitioned from a state marked by high expression of homeostatic chemokines to one characterized by upregulation of IFN-driven inflammatory genes, including *CXCL9*, *CXCL10* and *CXCL11*—known drivers of inflammatory immune cell recruitment (see above; Fig. 3a). In line with this, DLBCL-induced *CXCL9*, *CXCL10* and *CXCL11* expression in BECs was restricted to a previously described cellular subset of high endothelial venules, a known site of immune cell trafficking^9^ (Extended Data Fig. 6d).

**Extended Data Fig. 7:**
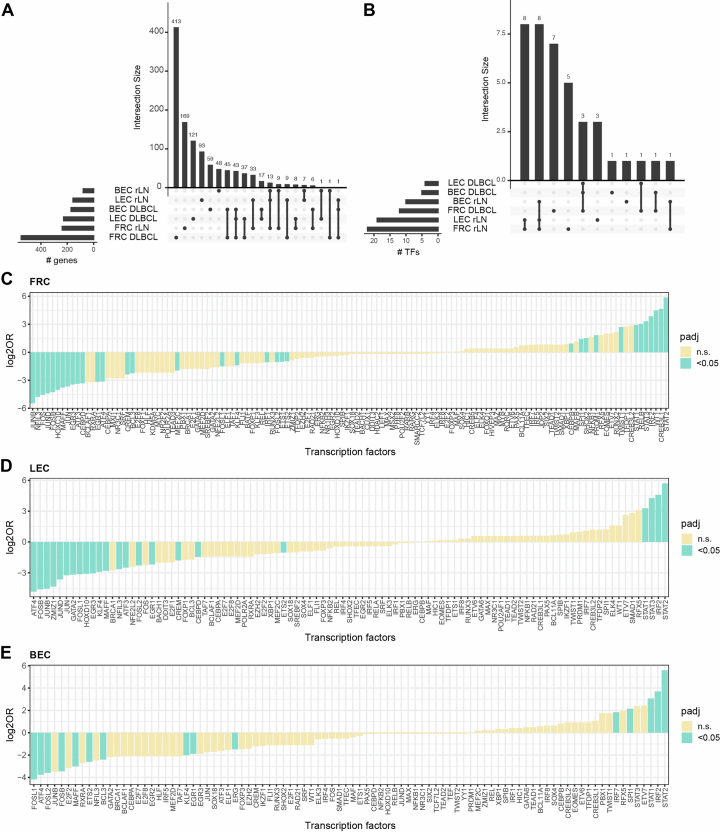
Differential transcription factor activity analysis using SCENIC. (**A**) Upset plot depicting the number and the overlap of significantly differentially expressed genes between rLN and DLBCL (p.adj < 0.05; log2FC > 1) used for differential transcription factor activity analysis. (**B**) Upset plot showing the number and the overlap of differentially active transcription factors between rLN and DLBCL (p.adj < 0.05; log2FC > 0.5). (**C**–**E**) Bar graphs depicting the log2OR calculated using two-sided Fisher’s test for the 3 stromal populations. Cyan color indicates p.adj < 0.05 after FDR correction. P-values were adjusted according to Benjamini-Hochberg. Source data

To uncover potential transcriptional regulators mediating stromal cell reprogramming and the dysregulation of chemokine expression in DLBCL-LNs, we determined gene regulatory networks using SCENIC^23^ and quantified the enrichment of differentially expressed target genes for each transcription factor (Methods). These analyses revealed well-characterized IFN-inducible transcription factors, such as *IRF2*, *IRF7*, *STAT1* and *STAT2*, as potential regulators of inflammatory chemokines, and unveiled putative regulators of homeostatic chemokine expression in LN stromal cells, including *EGR1*, which has previously been linked to mesenchymal cell function^24,25^ (Fig. 3b,c and Extended Data Fig. 7).

**Extended Data Fig. 8:**
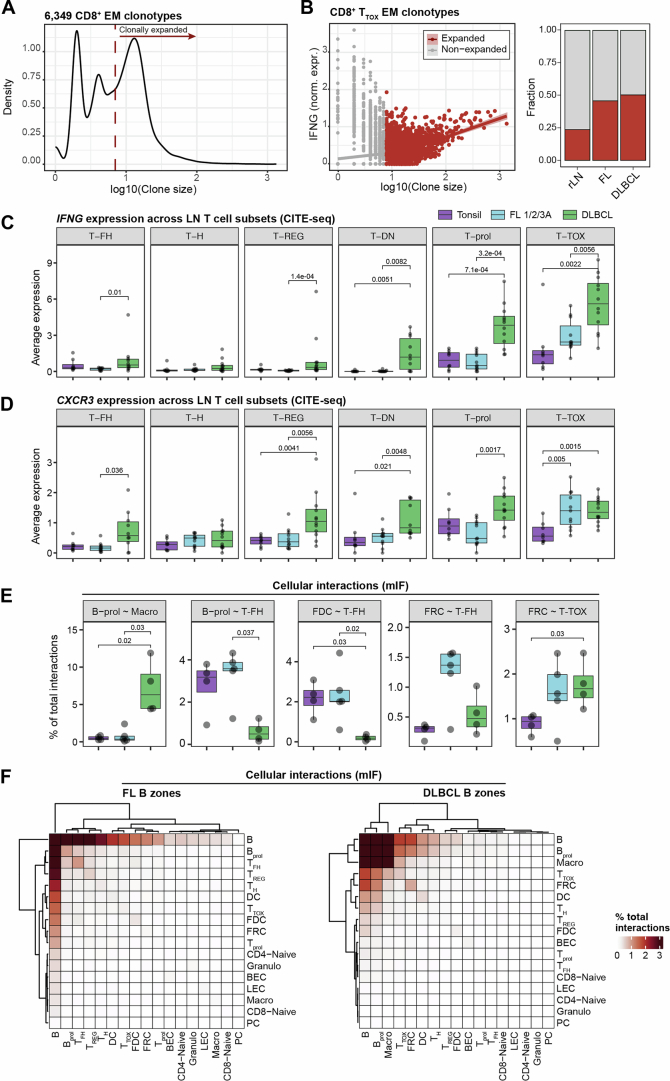
Interferon signaling determines the inflammatory microenvironment in DLBCL. (**A**) Density plot illustrating the clone size (log10 transformed) of 6,349 clonotypes as determined by single-cell T cell receptor profiling^12^. Vertical red line indicates the threshold above which a clone was considered clonally expanded. (**B**) Scatter plot showing the normalized expression of *IFNG* plotted against the log10 clone size. Red points are considered clonally expanded based on A (left panel). Bar plot showing the fraction of clonally expanded T cells across disease entities (right panel). (**C**, **D**) Box plots depicting average expression of *IFNG* (C) and *CXCR3* (D) across T cell subsets as measured by CITE-seq^12^. Data points represent individual patient samples. P-values were calculated using a two-sided unpaired Wilcoxon rank-sum test and adjusted according to Benjamini–Hochberg. (**E**) Box plots showing the percentage of total interactions per sample for representative cell-cell pairs within the mIF dataset. Cell-cell interactions were defined by the *k* = 6 nearest neighbors per cell, assuming a hexagonal cell shape. Data points represent individual patient samples. P-values were calculated using a two-sided unpaired Wilcoxon rank sum test. (**F**) Heatmap showing the overall cellular interaction landscape as determined by k nearest neighbor analysis (k = 6, as in (E)) in the B cell-rich zones (follicles and B_prol_ neighborhood) in FL (left) and DLBCL samples (right) within the mIF dataset. For panels **A**, **B**: rLN n = 3, FL n = 5 and DLBCL n = 3 patient samples. For panels **C**, **D**: rLN n = 8; FL n = 11; DLBCL n = 12 patients. For panels **E**, **F**: rLN n = 4; FL n = 5; DLBCL n = 4 patients). Box plots: center line: median; box: interquartile range, whiskers: 1.5x interquartile range. Source data

Consistent with increased IFN-induced activity in DLBCL FRCs, *IFNG* expression levels and the number of tumor-reactive, clonally expanded *IFNG*^+^ CD8^+^ T cells gradually increased from rLN to FL and DLBCL, providing an explanation for the extensive IFN-driven microenvironmental reprogramming in this entity (Fig. 3d–g and Extended Data Fig. 8a–c).

Cell–cell communication analysis using NicheNet^26^ between T cells and FRCs in our scRNA-seq data suggested an inflammatory feedback loop beginning with IFNγ-secreting tumor-reactive T cells that induce the expression of *CXCL9*, *CXCL10* and *CXCL11* in FRCs. These FRCs, in turn, chemotactically attract CXCR3^+^ T cells, further fueling *CXCL9*, *CXCL10* and *CXCL11* expression (Fig. 3h and Supplementary Table 7). Consistent with this, higher infiltration of *CXCR3*^+^ effector T cells was observed in FL-LNs and, to a greater extent, DLBCL-LNs, confirming the link between IFN-inducible chemokines produced by FRCs and the recruitment of inflammatory T cells through the CXCR3 receptor (Fig. 3i and Extended Data Fig. 8d). *CXCR3*^+^ T cells were located in closer proximity to *CXCL9*^+^ FRCs in DLBCL, consistent with increased inflammation-driven chemoattraction (Fig. 3j and Extended Data Fig. 8e). Notably, both cytotoxic T cells and FRCs concurrently increased in areas containing proliferating B cells, including the follicles in FL and the B_prol_ neighborhood in DLBCL, suggesting a close relationship between malignant B cell proliferation and inflammatory microenvironmental reprogramming (Extended Data Fig. 8e,f). Similarly, B_prol_ neighborhoods in DLBCL also showed infiltration by myeloid cells, including macrophages and dendritic cells, further corroborating the inflammatory state of the DLBCL microenvironment (Extended Data Fig. 8e,f).

To experimentally validate that cellular interactions with lymphocytes drive inflammatory reprogramming in LN stromal cells, we performed coculture experiments using primary lymphoid cells and DLBCL-derived immortalized FRCs (iFRCs), followed by scRNA-seq (Fig. 3k) and secretome analysis of the supernatant. Indeed, iFRCs underwent pronounced transcriptomic changes after exposure to DLBCL-derived lymphocytes, resembling the T cell-driven IFNγ-induced inflammatory phenotype switch observed in DLBCL-LNs in vivo (Fig. 3l,m). Moreover, upon coculture, an increase in inflammatory chemokines and a reduction in homeostatic chemokines were observed in the culture supernatants, confirming that cellular interactions with lymphoma-derived lymphocytes drive the reprogramming of FRCs (Fig. 3n and Supplementary Table 8).

Taken together, these findings suggest that the spatial disorganization of lymphomatous LNs is driven by an inflammatory feedback loop fueled by IFNγ-secreting tumor-reactive T cells, which reprogram LN stromal cells from a functional tissue-organizing state to an inflammatory, dysfunctional state.

### Chemokine-based in silico reconstruction of LN organization

**Fig. 4:**
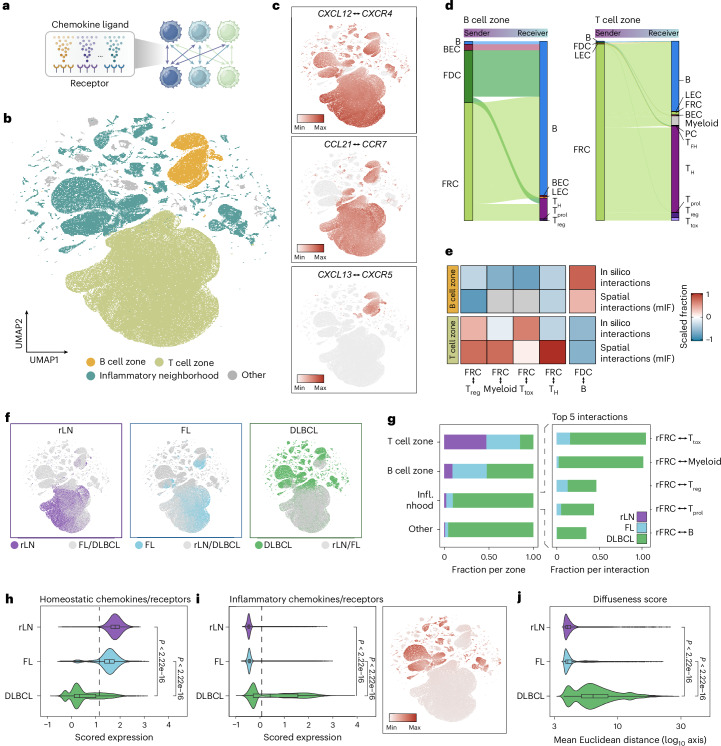
Cell–cell chemokine attraction potential recapitulates LN organization. **a**, Schematic overview of scRNA-seq-based in silico interaction analysis: the geometric mean for all possible cell combinations is calculated using chemokine ligand–receptor expression as input data. **b**, UMAP embeddings of cell–cell attraction potentials, where each dot represents a cell pair; cell pairs cluster based on their chemokine ligand–receptor expression. Clusters are annotated according to the respective zones. **c**, UMAPs colored by exemplary zone-defining ligand–receptor pairs. **d**, Alluvial plot of sender and receiver cell type frequencies in rLN samples. Minor cell type contributions below the 50th percentile were filtered out for representation. **e**, Scaled fraction of cell type frequencies comparing in silico and spatially mapped interactions in rLN samples from mIF data. **f**, UMAPs colored by disease entities. **g**, Left, relative contribution of each disease entity to the annotated in silico neighborhoods and zones. Right, top five main contributing cell–cell interaction pairs within the inflammatory neighborhood. **h**,**i**, Scored expression of homeostatic (**h**) and inflammatory (**i**, left) ligand–receptor pairs, along with a UMAP colored by the scored expression of inflammatory ligand–receptor pairs (**i**, right). Dashed lines indicate the mean across disease entities. *P* values were calculated using a two-sided Wilcoxon rank-sum test and adjusted using the Benjamini–Hochberg method. **j**, Violin plot representing the diffuseness score (mean Euclidean distance of cells in a high-dimensional principal component space) across entities (rLN *n* = 17,813, FL *n* = 16,704, DLBCL *n* = 28,921 interaction pairs). *P* values were calculated using a two-sided Wilcoxon rank-sum test and adjusted using the Benjamini–Hochberg method. For panels **b**–**i**, analyses were based on scRNA-seq-derived cell–cell interaction pairs (rLN *n* = 83,570, FL *n* = 90,087, DLBCL *n* = 116,536 interaction pairs). Box plots show the median (center line), the interquartile range (box) and whiskers extending to 1.5× the interquartile range; outliers are not displayed. Infl. nhood, inflammatory neighborhood. Illustration in **a** created in BioRender; Mathioudaki, A. https://biorender.com/jb5q3oh (2025). Source data

**Extended Data Fig. 9:**
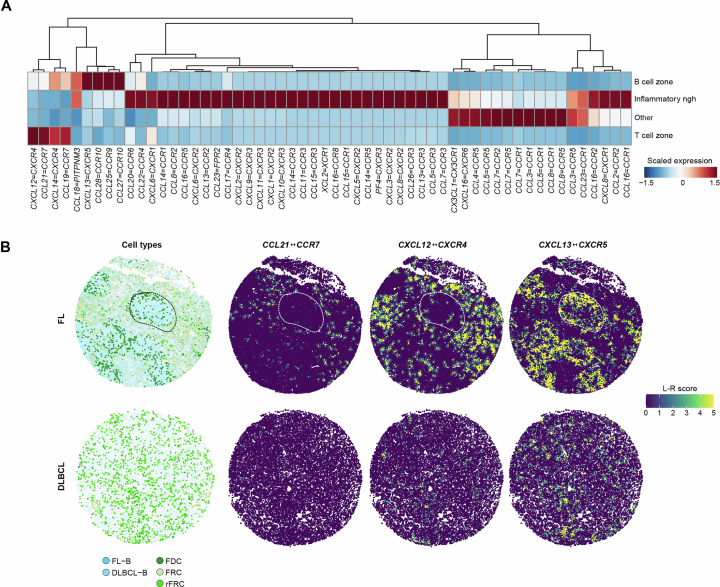
Chemokine expression across *in silico*- and spatially-defined LN compartments. (**A**) Heatmap colored by the scaled geometric mean expression of chemokines and their receptors per zone (as defined in Fig. 4). (**B**) Spatial transcriptomics plots of FL- (top panels) and DLBCL-LN (bottom panels) tissue cores colored by cell type (left panel) as well as ligand-receptor interaction scores (L-R scores) for zone-defining chemokine-receptor pairs (right panels). Dashed lines mark a representative B cell follicle. Source data

Our data suggest a model in which chemokine gradients, established by distinct subsets of stromal cells, coordinate the spatial self-organization of LNs, whereas inflammatory perturbations of these chemokine gradients in DLBCL underlie the loss of tissue architecture. To test this model, we investigated whether chemokine-based cell–cell attractions are sufficient to explain the organizational principles of LNs and their disruption in DLBCL. First, we defined the chemokine ‘attraction potential’ as the geometric mean expression of all chemokine receptors and their matching ligands (57 chemokine ligand–receptor pairs) across all possible cell pair combinations in our scRNA-seq data (Fig. 4a and Methods). Dimensionality reduction and clustering based on chemokine attraction potentials yielded a total of 63,438 sender–receiver cell pairs forming distinct clusters (Fig. 4b, Extended Data Fig. 9a and Methods)^27^. Importantly, several of the main clusters accurately recapitulated distinct spatio-organizational LN compartments, including their cellular composition, chemotactic attraction and underlying biology (Fig. 4b–d and Extended Data Fig. 9b). For example, one cluster recapitulated chemokine interactions and the cellular composition of T cell zones, including FRC-to-T cell interactions. Similarly, another cluster was dominated by *CXCL13*–*CXCR5* interactions and enrichment of *CXCL13*^+^ FDCs, thus recapitulating the cellular interactions of LN follicles (Fig. 4b–d).

Notably, within this framework, LN stromal cells were almost exclusively identified as chemokine senders, whereas hematopoietic cells were almost always identified as receivers, supporting the notion that stromal cells act as the main organizers of LN spatial architecture (Fig. 4d). We then investigated whether in silico*-*calculated chemokine attraction potentials of cell pairs predict spatial organization, by systematically comparing the frequencies of predicted cellular interactions with those measured in our mIF data (Fig. 4e). Furthermore, we calculated the ligand–receptor interaction scores of zone-defining chemokine–receptor pairs in our spatial transcriptomics data (Extended Data Fig. 9b). Together, these analyses revealed a high concordance between predicted and observed interactions, suggesting that chemokine-based cell attraction potentials act as major drivers in LN self-organization in humans, in line with mechanistic data from mouse models^13–21^.

Within rLNs and FL-LNs, the vast majority of chemokine-mediated cell–cell attraction potentials were confined to T cell and B cell zones, consistent with these two organizational structures dominating the observed spatial architecture (Fig. 4f,g). As expected, an increase in B cell zone interactions was observed in FL, reflecting the larger size and greater number of B cell follicles in this disease entity. Strikingly, in DLBCL-LNs, the two main clusters representing homogeneous B and T cell zone interactions were largely replaced by a heterogeneous mixture of many small clusters, characterized by interactions specifically involving dysfunctional rFRCs (Fig. 4f,g). To quantify the variability in chemokine-mediated cell–cell attraction potentials within LNs, we calculated a ‘diffuseness score’, defined as the average Euclidean distance between cell pairs in the principal component space (Methods). This analysis confirmed a shift from homogeneous chemokine-mediated cell–cell interactions in rLNs and FL-LNs to a heterogeneous spectrum of interactions characterized by a high diffuseness score in DLBCL and loss of homeostatic chemokine cell–cell interactions (Fig. 4h–j). In line with our hypothesis that inflammation-driven microenvironmental reprogramming underlies the loss of LN spatial organization, the fragmented clusters in DLBCL were largely characterized by IFN-inducible chemotactic interactions between *CXCL9*, *CXCL10* and *CXCL11* from stromal cells and *CXCR3*^+^ immune cells (Fig. 4h–j).

Together, these data demonstrate that chemokine-mediated cell–cell attraction potentials recapitulate the major organizational principles of LNs without prior knowledge of spatial information, predict their disruption in DLBCL and support a model in which inflammation-based environmental reprogramming contributes to the loss of tissue architecture.

### Homeostatic chemokines inform prognosis in B cell lymphoma

**Fig. 5:**
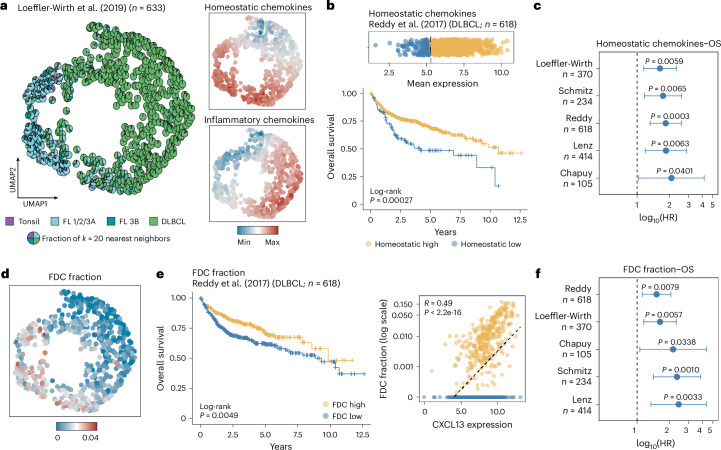
Changes in chemokine expression profiles are associated with worse overall survival in DLBCL. **a**, UMAP representation of a microarray dataset^22^ with homeostatic (*CXCL12*, *CXCL13*, *CCL19*, *CCL21*) and inflammatory (*CXCL9*, *CXCL10*, *CXCL11*) chemokine expression values used as features for dimensionality reduction. Left, UMAP displaying pie charts (within each dot) that represent the *k* = 20 nearest neighbors, colored according to disease entity. Right, the same UMAP colored according to the mean expression of homeostatic (top) and inflammatory (bottom) chemokines. **b**, Bulk RNA-seq dataset^29^ stratified according to homeostatic chemokine expression into high (*n* = 519 patients) and low (*n* = 99 patients) groups using maximally selected rank statistics, shown as a dot plot (top) and a Kaplan–Meier curve of overall survival (bottom). The *P* value was calculated using the log-rank test. **c**, Forest plot summarizing log_10_-transformed hazard ratios (center), 95% confidence intervals (error bars) and Wald-derived *P* values estimated from univariate Cox proportional hazards models assessing the association between homeostatic chemokine expression and overall survival across five individual DLBCL bulk datasets^22,28–31^. **d**, Same UMAP as in **a**, colored by CIBERSORTx-derived FDC fractions. **e**, Bulk RNA-seq dataset^29^ stratified by FDC abundance into high (*n* = 255 patients) and low (*n* = 364 patients) groups using maximally selected rank statistics based on CIBERSORTx fractions, shown as a Kaplan–Meier curve of overall survival (left; log-rank test) and a scatter plot of log-transformed FDC fractions and *CXCL13* expression (right; Pearson correlation). **f**, Forest plot summarizing log_10_-transformed hazard ratios (center), 95% confidence intervals (error bars) and Wald-derived *P* values estimated from Cox proportional hazards models assessing the association between FDC fraction and overall survival across five individual DLBCL bulk datasets^22,28–31^. For panels **a** and **d**: tonsil *n* = 10, FL 1/2/3A *n* = 145, FL 3B *n* = 48 and DLBCL *n* = 430 patients. OS, overall survival; HR, hazard ratio. Source data

In clinical practice, indolent FL and aggressive DLBCL are largely defined by their distinct morphologies. To test whether dysregulation of structure-defining chemokine expression profiles is sufficient to identify them as distinct entities, we determined homeostatic and inflammatory chemokine signatures as proxies for spatial organization in a large bulk transcriptomics dataset^22^. Notably, dimensionality reduction of chemokine expression patterns was sufficient to differentiate between indolent FL with a follicular growth pattern and aggressive DLBCL with a diffuse growth pattern (Fig. 5a), thus confirming the association between specific chemokine expression profiles and lymphoma architecture across a large patient cohort.

**Extended Data Fig. 10:**
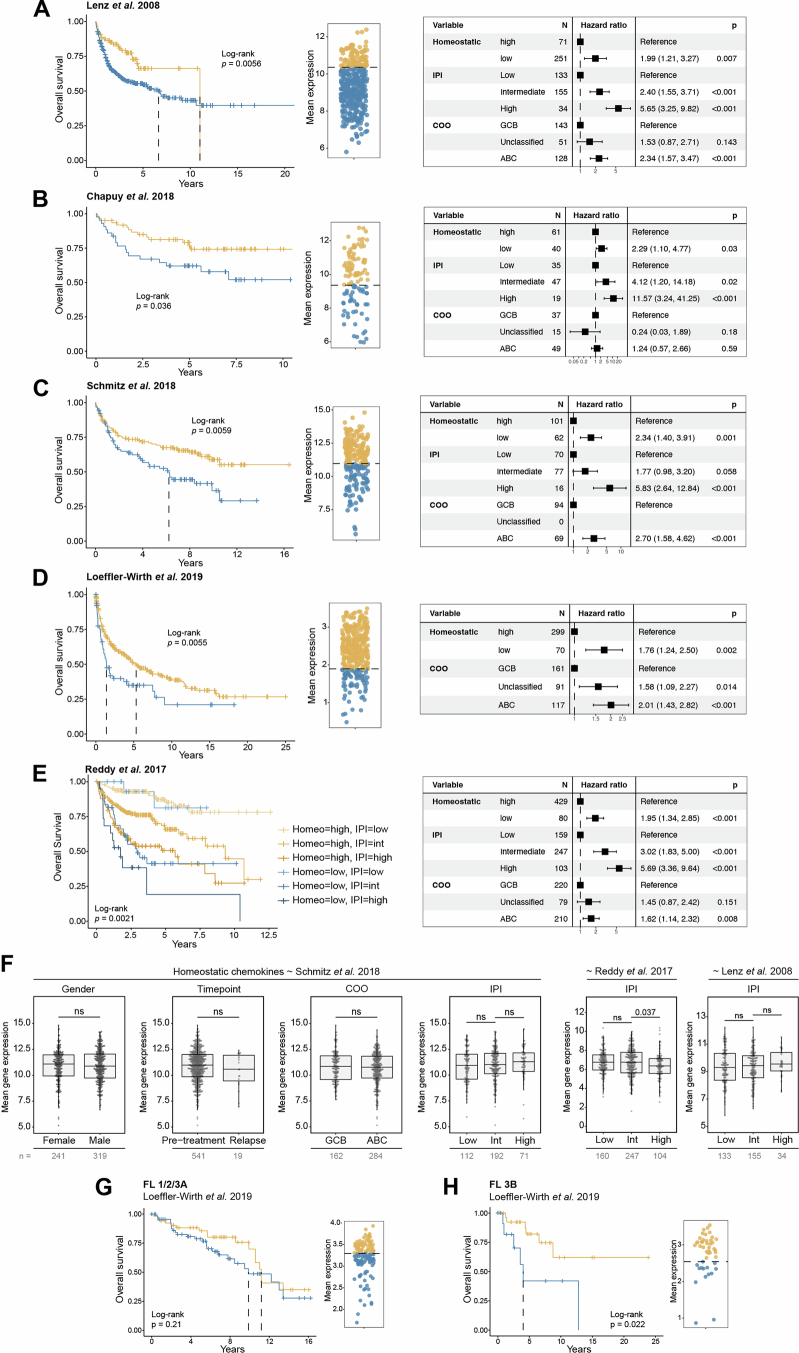
Attenuated homeostatic chemokine expression is linked to inferior overall survival in DLBCL and high-grade FL. (**A**–**D**) DLBCL samples from bulk transcriptomics cohorts^22,28,30,31^ were stratified into high and low expressers of homeostatic chemokines using maximally selected rank statistics (middle panels) and associations with overall survival are depicted in Kaplan-Meier curves (left panels). Forest plots visualize HRs (center), 95% confidence intervals (error bars) and Wald-derived p-values estimated from Cox proportional hazard models of homeostatic chemokine expression and overall survival, adjusted for COO and IPI (right panels). (**E**) Kaplan-Meier curve stratifying samples from the Reddy et al. cohort^29^ by homeostatic chemokine expression and IPI (left panel) and forest plot showing results from a Cox proportional hazard model (right panel), corresponding to Fig. 5b. (**F**) Box plots showing mean expression of homeostatic chemokines across patient metadata of exemplary bulk RNA-seq cohorts^28,29,31^. Boxes indicate the median (center line), interquartile range (bounds) and 1.5x interquartile range (whiskers). Data points represent patient samples. P-values were calculated using two-sided unpaired Welch’s t-test. (**G**, **H**) FL 1/2/3 A (A) and 3B (B) samples from bulk transcriptomics data^22^ were stratified by homeostatic chemokine expression into high and low samples using maximally selected rank statistics, represented in a dot plot (right panels) and Kaplan-Meier curve showing OS (left panels). P-values were calculated using log-rank tests. For panel G: high (orange) n = 57; low (blue) n = 73 patients. For panel **H**: high (orange) n = 32; low (blue) n = 14 patients. *Abbreviations:* COO = cell-of-origin, IPI = international prognostic index, ns = non significant, Homeo = homeostatic chemokines, HR = hazard ratio. Source data

Next, we investigated whether chemokine signatures are associated with clinical endpoints and whether they reflect patient heterogeneity within DLBCL. Indeed, low expression of homeostatic chemokines was associated with adverse overall survival in five independent cohorts of DLBCL bulk transcriptomics data (*n* = 1,726 patients in total)^22,28–31^ (Fig. 5b,c and Extended Data Fig. 10). Higher fractions of FDCs, as a surrogate for remnant *CXCL13* expression, translated into better overall survival across all DLBCL datasets (Fig. 5d–f and Extended Data Fig. 4e,f). Notably, homeostatic chemokine expression and survival associations were independent of clinical risk factors such as cell-of-origin annotation and the International Prognostic Index, suggesting spatial LN organization as an additional, orthogonal factor strongly linked to more aggressive disease biology in DLBCL (Extended Data Fig. 10a–f).

Together, these data support our finding that the aggressiveness of B cell lymphoma correlates with the gradual loss of LN spatial organization, which represents a crucial event in lymphomagenesis with a direct impact on patient outcomes.

## Discussion

In this study, we combined single-cell and spatially resolved mapping to investigate the architectural principles governing the organization of single cells into complex tissues, such as the LN, and the pathophysiological disruption of this organization during malignant transformation. Our results consolidate the central role of LN stromal cells in establishing chemokine gradients that jointly orchestrate the spatial architecture of the human LN, thereby validating mechanistic data from mouse models^13–21,32,33^. Importantly, we showcase how lymphoma-driven perturbations of chemokine networks may underlie disruptions in LN morphology as a functional consequence of DLBCL pathogenesis.

Our data suggest an inflammatory feedback loop fueled by IFNγ-producing tumor-reactive T cells that drives the reprogramming of LN stromal cells and renders them dysfunctional. In line with this, a recent study reported the reprogramming of LN stromal cells in DLBCL^34^. We demonstrate that LN stromal cells downregulate a range of homeostatic chemokines required to maintain functional LN architecture while upregulating IFN-induced chemokines. Similarly, during acute infections, IFNγ release mediates the downregulation of the homeostatic chemokines CXCL13 and CCL21, prompting a spatial rearrangement to minimize competition for resources during immune responses^35^. While IFNγ production is transient during infections, its constant production by tumor-reactive T cells in lymphoma may cause long-term LN architectural changes. Importantly, the transcriptional rewiring of LN stromal cells from a non-malignant state to FL and DLBCL mirrors the gradual loss of tissue architecture. The expression of homeostatic chemokines is partly maintained by region-specific FRC populations in FL but is completely abrogated in diffusely growing DLBCL.

Additionally, our data highlight the involvement of T cells in deregulating the LN chemokine equilibrium. Specifically, we identified lymphoma-induced ectopic expression of CXCL13 in diffusely distributed cytotoxic T cells. Although CXCL13^+^ cytotoxic T cells promote B cell recruitment in solid cancers^36,37^, they may also contribute to chemokine imbalances and structural loss in lymphomatous LNs. Besides the deregulation of chemokines involved in spatial LN organization, we observed a gradual increase in IFN-induced CXCL9, CXCL10 and CXCL11 production, as well as the chemotactic recruitment of CXCR3^+^ immune cells from non-malignant LNs to FL-LNs and DLBCL-LNs, further fueling the generation of an inflammatory milieu. IFNγ-producing tumor-reactive T cells were predominantly expanded in DLBCL, explaining the gradual increase in inflammation-based remodeling and FRC dysfunction. Jointly, our model suggests that inflammatory reprogramming of the LN microenvironment drives the dysregulation of structure-defining chemokine gradients, leading to the breakdown of LN architecture in DLBCL.

Conceptually, these findings are relevant in several respects. First, our results provide a mechanistic framework for the diffuse growth pattern in DLBCL, which is an important diagnostic criterion for distinguishing DLBCL from other, more indolent entities. Our study suggests that the loss of spatial organization in DLBCL is likely a consequence of active microenvironmental LN reprogramming rather than a mere result of passive overgrowth. Second, we demonstrate that alterations in LN chemokine networks and the associated architectural changes are key processes in lymphomagenesis. While functional LNs are fundamentally important for generating efficient antitumor immunity, the loss of LN functionality due to its spatial disruption may underlie cancer immune evasion. Finally, our results uncover and confirm the general principles of LN organization in humans, as previously described in mouse models^1,2^.

While previous studies have exploited single-cell and spatially resolved technologies to characterize tissue microenvironments, here we take an integrative approach to derive the principles governing LN tissue organization. By inferring the attraction of LN-resident cells based on their chemokine–receptor expression profiles, we accurately predicted spatial architectures and their disease-mediated destruction.

Together, our results provide detailed insights into the architectural principles of human LN organization and suggest how lymphoma-induced microenvironmental reprogramming drives the loss of tissue organization.

## Methods

### Collection of primary patient samples

All patient samples were collected from adult patients after obtaining written informed consent in accordance with the Declaration of Helsinki, including consent for the publication of deidentified data derived from these samples. The Ethics Committee of the Medical Faculty of the University of Heidelberg approved the study (S-057/2019). No compensation was provided to participants. Age, sex and gender were not included in the study design. No exclusions were made based on race, ethnicity, sex, gender, age or social factors. Sex was assigned based on clinical records. No sex- or gender-based analysis was performed. No statistical methods were used to predetermine sample sizes due to the exploratory nature of the study, but our sample sizes are similar to those reported in previous publications. Analyzed samples were fully consumed during data acquisition; additional aliquots from the same specimen are available upon reasonable request to the corresponding authors. Patient information is provided in Supplementary Table 1.

### Analysis of mIF data

mIF data on formalin-fixed paraffin-embedded tissues of rLN, FL-LN and DLBCL-LN samples were obtained from a previously published study^12^. Segmented single cells were further annotated with respect to known non-hematopoietic subsets: LECs (CD31^+^PDPN^+^), BECs (CD31^+^CD34^+^PDPN^−^), FDCs (CD21^+^PDPN^+^) and non-FDC FRCs (CD21^−^PDPN^+^CD90^+^). For each single cell across samples, we identified neighboring cells using the *k*-nearest neighbors algorithm (*k* = 20; Scikit-learn version 1.3.2)^38^. Single cells were then grouped into distinct unsupervised clusters using *k*-means clustering with *k* = 20 (Scikit-learn version 1.3.2)^38^. After binning redundant clusters with similar compositions, the clusters were further annotated to known LN compartments or regions based on the enrichment of specific cell types (log_2_(odds ratio)) as well as their localization. Marker positivity (for example, CXCL13^+^ cells) was determined by applying the Otsu thresholding method.

### Generation of LN-derived single-cell suspensions

Freshly excised human rLN, FL-LN and DLBCL-LN samples were placed in RPMI 1640 (Gibco) and cut into small pieces using surgical scalpels and scissors. Tissue pieces were dissociated using a combined mechanical and enzymatic protocol. Briefly, tissues were digested for three cycles of incubation with RPMI 1640 containing 0.8 mg ml^−1^ dispase (Sigma), 0.2 mg ml^−1^ collagenase P (Roche) and 0.1 mg ml^−1^ DNase I (Roche) for 20 min at 37 °C, followed by repetitive pipetting using a 1-ml Pasteur pipette. After each cycle, the enzyme mixture was replaced, and the collected single-cell suspensions were filtered through 100-μm strainers into calcium-free PBS containing 2% FBS and 5 mM EDTA to quench collagenase activity. Lymphoid and non-lymphoid fractions were isolated by combined labeling and column-free magnetic separation of B and T cells using the EasySep Human CD3 and CD19 Positive Selection Kits (STEMCELL Technologies). Positive (lymphoid) and negative (non-lymphoid) fractions were washed once in PBS, cryopreserved separately in FBS supplemented with 10% DMSO and stored in liquid nitrogen until further use. For downstream experiments, no randomization was performed, and all available samples were used.

### Cell sorting and single-cell transcriptomics

Lymphoid and non-lymphoid fractions were thawed and washed in RPMI 1640 containing 10% FBS. Cells were resuspended in fluorescence-activated cell sorting (FACS) buffer (PBS + 5% FBS + 0.5 mM EDTA), followed by staining for viability using Fixable Viability Dye eFluor 506 and calcein (Thermo Fisher Scientific), lymphocyte or stromal antibody panels, as well as hashtag antibodies using the BD Single-Cell Multiplexing Kit (BD Biosciences). The flow antibodies used in the study are listed in Supplementary Table 9. Cells were analyzed and sorted into a sample buffer (BD Rhapsody Cartridge Reagent Kit) using a FACSAria Fusion cell sorter (BD Biosciences) equipped with a 100-μm nozzle. Briefly, we established the following sorting gates: CD3^+^ T cells, CD19^+^ B cells, CD31^+^PDPN^−^ BECs, CD31^+^PDPN^+^ LECs, CD31^−^PDPN^+^ FRCs and CD31^−^PDPN^−^ double-negative stromal cells. The detailed sorting strategy is depicted in Extended Data Fig. 1c. All hematopoietic and non-hematopoietic populations were enriched to 2,000 cells per sorting gate. Sorted cells were pooled and captured using the BD Rhapsody single-cell system following the manufacturer’s instructions. Multiplexing and whole-transcriptome mRNA libraries were generated according to the library preparation protocols provided by the manufacturer (BD Biosciences). The resulting libraries were assessed using Qubit (Thermo Fisher Scientific) and Bioanalyzer (Agilent Technologies), pooled, and sequenced on the NextSeq 500 instrument (Illumina) in high-output mode.

### Processing and annotation of single-cell transcriptomics data

FASTQ files were processed using the BD Rhapsody docker image version 1.9 (https://hub.docker.com/u/bdgenomics) and the Common Workflow Language on a CentOS machine that meets the requirements specified by BD (BD Doc ID: 47383). The pipeline removed read pairs from R1 and R2 if the reads were too short (R1 < 66 base pairs (bp) and R2 < 64 bp) or of low quality (quality score < 20). Next, quality-filtered R1 reads were annotated to identify cell barcodes and unique molecular identifiers (UMIs), and high-quality R2 reads were aligned to the reference genome (GRCh38 GENCODE version 29) using Bowtie 2 (ref. ^39^). Information from R1 and R2 was combined, and UMIs were corrected using a recursive substitution error correction (RSEC) algorithm developed by the manufacturer. In all analyses, the RSEC-corrected counts-per-molecule matrices were used for further downstream analysis. Patient demultiplexing information was provided in a separate file as a sample tag per cell barcode, which was used as additional metadata in the analyses.

Counts-per-molecule information was loaded into R (version 4.1.0), and analyses were continued using Seurat (version 4.3.0)^40^. UMIs were log-normalized to account for differences in sequencing depth across all cells. Cells with a high feature count (>8,000), total counts (>75,000) and mitochondrial gene content (>25%) were removed. Batch correction was performed using Scanorama version 1.7.3 and the 3,000 most variable genes^41^. The corrected counts were used to calculate a shared nearest neighbor graph and to perform Louvain clustering (resolution factor 1.4). The optimal cluster resolution was assessed using Clustree^42^. Graphical visualization by UMAP was generated using 30 neighboring points in the local approximation of the manifold structure. Features of the first 45 principal components, determined from the Scanorama-corrected count data, were used for the UMAP. For all other downstream analyses (for example, differential gene expression analysis, interaction analysis and clustering), the log-normalized raw counts were used. Differentially expressed genes per cluster were determined using the FindAllMarkers function from Seurat^43^. Clusters with similar marker profiles were merged and annotated according to their respective cell types. rBEC and rFRC were defined as clusters with a contribution of DLBCL-derived cells greater than 90% (Extended Data Fig. 2a). Reported expression scores were computed using Seurat’s AddModuleScore function.

### Differential expression and transcription factor activity analysis

Differential gene expression between conditions was assessed using Seurat’s FindMarkers function on the RNA assay with the MAST (version 1.22.0) method, which is suitable for single-cell transcriptomics data^44^. The analysis identified differentially expressed genes between conditions in all cell populations (adjusted *P* < 0.05 and log_2_(fold change) > 0.5; *P* values were adjusted for multiple comparisons using the Bonferroni correction method).

Functional enrichment analysis of differentially expressed genes was performed using the ClusterProfiler package (version 4.6.3)^45^. The specific functions applied were enrichGO for Gene Ontology enrichment analysis, compareCluster for Kyoto Encyclopedia of Genes and Genomes (KEGG) pathway analysis, the enrichr function for the Molecular Signatures Database (MSigDB)^46,47^ (Hallmark collection) and enrichPathway for pathway annotation from ReactomePA^48^. Default settings were used, and all expressed genes in the dataset served as the background gene set. *P* values were adjusted using the Benjamini–Hochberg method, with a cutoff set at 0.05.

The pySCENIC workflow^23^ was run using an in-house Snakemake pipeline. Gene regulatory network inference was performed with the GRNBoost2 algorithm from the Arboreto package, using 50 perturbations and splitting the dataset in half^49^. The analysis was performed on the raw data. The human v9 motif collection from cisTarget (hg38__refseq-r80__10kb_up_and_down_tss.mc9nr.feather, hg38__refseq-r80__500bp_up_and_100bp_down_tss.mc9nr.feather) was used to identify transcription factor regulons. For the AUCell scoring, target genes occurring in more than 40 out of 100 runs were considered. The resulting area-under-the-curve matrices were used for visualization and downstream analysis. For the finalized gene regulatory network, target genes present in more than 80% of the runs were included. Differential activity of transcription factors was assessed using the SCENIC-derived gene regulatory network. SCENIC provides regulons and per-cell inferred transcription factor activities. Differentially active transcription factors were detected using Fisher’s exact test to assess the enrichment of condition-specific differentially expressed genes among all target genes (adjusted *P* < 0.05 after Bonferroni correction).

### Cell–cell communication analysis

In silico attraction potential analysis of scRNA-seq data was performed using Scriabin^50^. For inferring cell–cell communication, only chemokines as ligands and their corresponding receptors were considered. Chemokine ligand–receptor information was extracted from the CytoSig database, which contains 63 chemokine-related ligand–receptor pairs^51^. Each cell within the dataset was considered either a receiver or a sender. Based on the geometric mean expression of each ligand–receptor pair for all possible cell–cell combinations within a single sample, Scriabin generates a cell–cell communication matrix. The output matrix was filtered to include at least four ligand–receptor pairs per cell–cell combination. Subsequently, the data were represented in a UMAP using 30 nearest neighbor points, 20 principal components and the cosine distance measure. The geometric mean expression values of ligand–receptor pairs were treated as new features, and cell–cell pairs were visualized. The attraction potential was calculated for each sample individually, and the samples were then merged for downstream analysis.

To calculate the diffuseness score, we computed the Euclidean distance in the principal component space after randomly downsampling cell–cell pairs to 30% of their original size. Distances were averaged per patient across cell–cell pairs from each disease entity and represented as continuous scores.

To predict ligand–receptor links between interacting cell types more generally, we used NicheNet’s (version 1.1.1) nichenet_seuratobj_aggregate function on the RNA assay (expression_pct = 0.05, lfc_cutoff = 0.2)^26^. Prior models for ligand–target, ligand–receptor and weighted network matrices were provided by NicheNet.

### Analysis of T cell clonality

T cell clonality analysis was performed on our previously published single-cell T cell receptor sequencing data^12^. In short, 5′ scRNA-seq data were acquired from DLBCL (*n* = 3), FL (*n* = 5) and rLN (*n* = 3) patient samples (75,054 single cells). These data enabled the identification of full-length T cell receptor sequences and, subsequently, clonally expanded T cells. T cell clonotypes were defined as clonally expanded when detected in more than seven cells. To further characterize clonally expanded T cell subsets, we mapped the 5′ scRNA-seq data to CITE-seq (cellular indexing of transcriptomes and epitopes by sequencing) reference data^12^ using the FindTransferAnchors and MapQuery functions from Seurat^43^. This integration enabled the phenotypic annotation of T cells from the 5′ dataset using labels derived from the CITE-seq reference, which combines transcriptomic and surface protein-level information. Protein markers captured using CITE-seq allow for more accurate identification of functionally distinct T cell states that may not be well resolved by transcriptomic data alone.

### Spatial transcriptomics data acquisition and analysis

Tissue sections of FL (*n* = 1) and DLBCL (*n* = 1) formalin-fixed paraffin-embedded LN tissue cores were prepared at a thickness of 5 μm. Spatial transcriptomics data were acquired using the Xenium Prime 5k gene panel with a 96-gene custom add-on panel (10x Genomics; instrument software version 3.1.0.0, analysis version xenium-3.1.0.4). Downstream analysis was restricted to nuclear transcripts and performed using Seurat^43^ (version 4.3.0). After filtering (retaining cells with >50 transcripts per cell), count data were normalized using SCTransform, and dimensionality reduction and clustering were performed using standard Seurat pipelines. Cluster-level cell type annotation was performed using scRNA-seq-informed markers. Neighborhood-based ligand–receptor scores of chemokines and their corresponding receptors were calculated based on pairwise Euclidean distances, considering neighbors within a 20-μm radius. Scores represent the number of neighboring cell pairs with ligand–receptor expression (and vice versa). To quantify the chemoattraction of *CXCR3*^+^ T cells by *CXCL9*^+^ FRCs (defined by normalized expression values > 2), we performed spatial point pattern analysis using the spatstat package (version 3.0.8)^52^. Kernel density estimations of *CXCR3*^+^ T cell nuclei centroid coordinates were computed, and local densities were then interpolated at the positions of *CXCL9*^+^ and *CXCL9*^−^ FRCs.

### Analysis of bulk transcriptomics data

For validation in independent cohorts, we assembled five previously published and clinically well-annotated bulk transcriptomics datasets, including a cohort of FL, DLBCL and healthy control tonsil samples^22^, as well as four DLBCL cohorts^28–31^. In microarray datasets, probes were translated into gene symbols using the hgu133a.db package (version 3.13), retaining the probe with the highest median intensity when multiple probes matched one gene symbol.

Cell type abundances in bulk datasets were deconvoluted using CIBERSORTx^53^. Single-cell reference profiles (signature matrix) were computed from our scRNA-seq data using the FindAllMarkers function from Seurat^43^, followed by output filtering (adjusted *P* < 0.01 and log_2_(fold change) > 1), retaining the top 100 markers by log_2_(fold change) per cell population and reducing redundancy by excluding highly correlated features (pairwise absolute correlation cutoff > 0.9). CIBERSORTx was run separately for each dataset using 500 permutations for significance analysis.

For survival analyses, we calculated the mean expression of homeostatic chemokines (*CXCL12*, *CXCL13*, *CCL19*, *CCL21*) or used CIBERSORTx-derived FDC fractions. Samples were stratified into ‘high’ and ‘low’ groups using maximally selected rank statistics from the maxstat^54^ package (version 07.25), as implemented in the surv_cutpoint function from the survminer^55^ package (version 0.5.0). Kaplan–Meier curves were fitted using the survfit function from the survival^56^ package (version 3.7.0), with the log-rank test used for *P*-value estimation. Cox proportional hazards regression models were fitted using the coxph function from the survival^56^ package.

### Quantification of chemokine levels in peripheral blood plasma

Peripheral blood samples were collected in EDTA S-Monovette tubes (Sarstedt) from 40 treatment-naive patients with FL (*n* = 18) or DLBCL (*n* = 22). Within 2 h of collection, the samples were centrifuged for 10 min at 2,000*g*. Plasma was snap-frozen in liquid nitrogen, following a previously established protocol^57^. Plasma samples were stored at −80 °C until analysis. Absolute CXCL13 plasma levels were quantified in duplicates using the Human CXCL13 Quantikine ELISA Kit (R&D Systems) according to the manufacturer’s instructions. Absorbance was measured using a FLUOstar Omega microplate reader (BMG Labtech) and the associated software (Omega version 6.2, BMG Labtech).

### In vitro coculture experiments

iFRCs were generated from primary LN-derived stromal cells isolated from a DLBCL-LN patient sample. Briefly, LN non-hematopoietic fractions were cultured in RPMI 1640 supplemented with 10% FBS, 1% penicillin–streptomycin and 2 mM l-glutamine. Following the outgrowth of primary mesenchymal cells in culture, the cells were lentivirally immortalized with specific transgene combinations and expanded in vitro (InSCREENeX)^58^. A stable FRC-like phenotype of expanded cells was confirmed by staining for mesenchymal markers using immunofluorescence imaging and FACS.

For coculture assays of iFRCs and primary LN-derived lymphocytes, FRCs were pre-plated at 4 × 10^4^ cells per ml in RPMI 1640 supplemented with 10% FBS, 1% penicillin–streptomycin and 2 mM l-glutamine, then incubated for 8 h to allow cell adherence. Next, LN single-cell suspensions were thawed and washed in RPMI 1640 containing 10% FBS, and lymphoid fractions were magnetically separated using EasySep Human CD3 and CD19 Positive Selection Kits (STEMCELL Technologies). Respective cell fractions were seeded at 5 × 10^5^ cells per ml on top of pre-plated iFRCs.

For scRNA-seq, iFRCs and LN-derived B and/or T cells from DLBCL samples (*n* = 3) were collected after 24 h of incubation, and different experimental conditions were multiplexed using in-house cell multiplexing oligonucleotides (a full list of barcodes is provided in Supplementary Table 9). Cells were incubated with cell multiplexing oligonucleotides at a final concentration of 1.8 μM for 20 min on ice, followed by four washes with PBS (centrifugation at 400*g* for 3 min at 4 °C). scRNA-seq and multiplexing libraries were prepared using the Single Cell 3′ Gene Expression v4 assay (10x Genomics) according to the manufacturer’s protocol. Sequencing was performed on a NovaSeq 6000 platform (Illumina) using paired-end 100-bp reads on an S4 flow cell. Data were preprocessed using Cell Ranger version 8.0.1.

For secretome profiling, supernatants from iFRCs cultured alone or in combination with primary LN-derived B and T cells (rLN *n* = 3; FL *n* = 3; DLBCL *n* = 3) were collected after 48 h of incubation. Protein levels were determined using the scioCD antibody microarray, which covers 119 cytokines and chemokines (Sciomics). Following spot segmentation, median signal intensities were normalized using an invariant LOWESS (locally weighted scatterplot smoothing) method. Full results are provided in Supplementary Table 8. All experiments included an autologous control of DLBCL-derived lymphocytes and iFRCs from the same LN sample.

### B cell migration assay

Primary LN single-cell suspensions were thawed and washed in RPMI 1640 containing 10% FBS. Cells were resuspended in FACS buffer (PBS + 5% FBS + 0.5 mM EDTA), followed by staining for viability and lymphocyte markers (Supplementary Table 9). After washing, cells were analyzed and sorted into RPMI 1640 without FBS using a FACSAria Fusion cell sorter (BD Biosciences) equipped with a 70-μm nozzle. Sorted cells were seeded in triplicate into the top chambers of 96-well HTS Transwell plates with 8-μm pores (Corning), with the bottom chambers containing either 1 μg ml^−1^ human recombinant CXCL13 (cat. no. 801-CX-025, R&D Systems) or PBS as a control. After 4 h of incubation at 37 °C, the Transwell inserts were removed. Cells were then transferred to 96-well V-bottom plates (Corning), washed twice and restained using the same flow antibody panel. Following the addition of Precision Count Beads (BioLegend), samples were measured using a Cytek Aurora Spectral Flow Cytometer (Cytek Biosciences). The absolute numbers of migrated B cells per well were calculated as follows: B cell count/bead count × total bead concentration.

### Statistics and reproducibility

No statistical method was used to predetermine the sample size. No data were excluded from the analyses. Data collection and analysis were not performed blinded to the conditions of the experiments. Statistical tests used for individual analyses are described in the figure legends and the Methods.

### Reporting summary

Further information on research design is available in the Nature Portfolio Reporting Summary linked to this article.

## Supplementary information

Reporting Summary

Supplementary Tables Supplementary Tables 1–9.

## Source data

Source Data Fig. 1 Statistical source data.

Source Data Fig. 2 Statistical source data.

Source Data Fig. 3 Statistical source data.

Source Data Fig. 4 Statistical source data.

Source Data Fig. 5 Statistical source data.

Source Data Extended Data Fig. 1 Statistical source data.

Source Data Extended Data Fig. 2 Statistical source data.

Source Data Extended Data Fig. 3 Statistical source data.

Source Data Extended Data Fig. 4 Statistical source data.

Source Data Extended Data Fig. 5 Statistical source data.

Source Data Extended Data Fig. 6 Statistical source data.

Source Data Extended Data Fig. 7 Statistical source data.

Source Data Extended Data Fig. 8 Statistical source data.

Source Data Extended Data Fig. 9 Statistical source data.

Source Data Extended Data Fig. 10 Statistical source data.

## Data Availability

Objects used for figure generation are available on Zenodo (10.5281/zenodo.18474620)^59^. FASTQ files of scRNA-seq data are available in the European Genome–phenome Archive (EGA) under submission numbers EGAS00001006986 and EGAS50000001252. Access to these data is controlled because they contain genomic information. Qualified researchers may request access through the EGA controlled-access system by submitting a data access application. Requests are evaluated for compliance, and applicants are typically notified of the decision within 2–4 weeks. Previously published mIF, CITE-seq and T cell receptor sequencing data that were reanalyzed here are available under accession codes S-BIAD565 (BioStudies) as well as GSE252608 and GSE252455 (ref. ^12^). Previously published microarray and RNA-seq data that were reanalyzed here are available under accession codes GSE22470, GSE48184, GSE43677 and GSE103944 (ref. ^22^); GSE10846 (ref. ^28^); EGAS00001002606 (ref. ^29^); GSE98588 (ref. ^30^); as well as phs001444, phs000178 and phs001184 (ref. ^31^). Source data are provided with this paper.

## Code availability

All custom code used for figure generation is publicly available on GitHub at https://github.com/ZauggGroup/LNarch.
