# Supplementary material for: Reprogramming of stroma-derived chemokine networks drives the loss of tissue organization in nodal B cell lymphoma
Source: Nat Cancer. 2026 Mar 25;7(3):538–52. doi: 10.1038/s43018-026-01136-z (PMC13035477; doi:10.1038/s43018-026-01136-z)
Supplement: Supplementary file 1 — Reporting Summary [file 43018_2026_1136_MOESM1_ESM.pdf]

nature portfolio

Corresponding author(s): Sascha Dietrich
Simon Haas
Daniel Hübschmann
Judith B. Zaugg

Last updated by author(s): Jul 13, 2024

Reporting Summary

Nature Portfolio wishes to improve the reproducibility of the work that we publish. This form provides structure for consistency and transparency in reporting. For further information on Nature Portfolio policies, see our Editorial Policies and the Editorial Policy Checklist.

Statistics

For all statistical analyses, confirm that the following items are present in the figure legend, table legend, main text, or Methods section.

n/a	Confirmed	
☐	☒	The exact sample size (*n*) for each experimental group/condition, given as a discrete number and unit of measurement
☐	☒	A statement on whether measurements were taken from distinct samples or whether the same sample was measured repeatedly
☐	☒	The statistical test(s) used AND whether they are one- or two-sided<br>*Only common tests should be described solely by name; describe more complex techniques in the Methods section.*
☐	☒	A description of all covariates tested
☐	☒	A description of any assumptions or corrections, such as tests of normality and adjustment for multiple comparisons
☐	☒	A full description of the statistical parameters including central tendency (e.g. means) or other basic estimates (e.g. regression coefficient) AND variation (e.g. standard deviation) or associated estimates of uncertainty (e.g. confidence intervals)
☐	☒	For null hypothesis testing, the test statistic (e.g. *F*, *t*, *r*) with confidence intervals, effect sizes, degrees of freedom and *P* value noted<br>*Give P values as exact values whenever suitable.*
☒	☐	For Bayesian analysis, information on the choice of priors and Markov chain Monte Carlo settings
☒	☐	For hierarchical and complex designs, identification of the appropriate level for tests and full reporting of outcomes
☒	☐	Estimates of effect sizes (e.g. Cohen's *d*, Pearson's *r*), indicating how they were calculated

Our web collection on statistics for biologists contains articles on many of the points above.

Software and code

Policy information about availability of computer code

Data collection	Single cell data was acquired using the BD Rhapsody single-cell system and associated software (BD Biosciences) or 10x Genomics Single Cell 3-prime Gene Expression v4 assays (10x Genomics) following the manufacturer's instructions. Sequencing was performed using NextSeq 500 or NovaSeq 6000 platforms (Illumina). Fastq files were processed with the BD Rhapsody Docker image version 1.9 (https://hub.docker.com/u/bdgenomics) and Common Workflow Language (CWL) on a Centos machine fulfilling the requirement by BD (BD Doc ID: 47383). Sequence alignment was performed using Bowtie2.<br>Spatial transcriptomics data was acquired using the 10X Xenium Prime 5k gene panel with a 96-gene custom add-on panel (instrument software version 3.1.0.0, analysis version xenium-3.1.0.4).<br>For the acquisition of flow cytometry data, we used a FACSAriaTM Fusion cell sorter (BD Biosciences) as well as FACSDiva software. ELISA assays were performed with the indicated commercial kits using a FLUOstar Omega microplate reader (BMG Labtech) and associated software (Omega version 6.2, BMG Labtech)
Data analysis	Data were analyzed in R v4.1.0 using the packages Seurat v4.3, MAST v1.22, ggplot2 v3.2, clusterProfiler v4.6.3, NicheNet v1.1.1, biomaRt v2.28, survival v3.1.12, survminer v0.4.9, maxstat v.07.25, hgu133a.db v3.13, spatstat v3.0.8, ComplexHeatmap v2.14 and autothresholdr v1.4 as well as Python v3.10 using the packages Scikit-learn v.1.3.2, pySCENIC v0.12.1 and Scanorama v16. A full description of data analysis is contained in the Methods section.<br>Scripts used for figure generation are available in https://github.com/ZauggGroup/LNarch.

For manuscripts utilizing custom algorithms or software that are central to the research but not yet described in published literature, software must be made available to editors and reviewers. We strongly encourage code deposition in a community repository (e.g. GitHub). See the Nature Portfolio guidelines for submitting code & software for further information.

Data

Policy information about availability of data

All manuscripts must include a data availability statement. This statement should provide the following information, where applicable:

- Accession codes, unique identifiers, or web links for publicly available datasets
- A description of any restrictions on data availability
- For clinical datasets or third party data, please ensure that the statement adheres to our policy

Objects used for figure generation are available at Zenodo (https://zenodo.org/records/18474620). FASTQ files of scRNA-seq data are available in the European Genome-Phenome Archive (EGA) under submission numbers EGAS00001006986 and EGAS50000001252. Access to these data is controlled since they contain genomic information. Qualified researchers may request access through the EGA controlled-access system by submitting a data access application. Requests are evaluated for compliance, and applicants are typically notified of a decision within 2–4 weeks.
Source data are provided with this paper.
Previously published mIF, CITE-seq and TCR-seq data that were re-analysed here are available under accession codes S-BIAD565 (BioStudies) as well as GSE252608 and GSE252455 (ref. 12).
Previously published microarray and RNA-seq data that were re-analysed here are available under accession codes GSE10172, GSE22470, GSE48184, GSE43677 and GSE103944 (ref. 22), GSE10846 (ref. 28), EGAS00001002606 (ref. 29), GSE98588 (ref. 30), as well as phs001444 and phs001184 (ref. 31).

Research involving human participants, their data, or biological material

Policy information about studies with human participants or human data. See also policy information about sex, gender (identity/presentation), and sexual orientation and race, ethnicity and racism.

Reporting on sex and gender	Age, sex or gender were not included in the study design. No exclusions were made based on race, ethnicity, sex, gender, age or social factors. Sex was assigned based on clinical records. No sex- or gender-based analysis was performed.<br><br>Total sample numbers:<br>- mIF: female n=2, male n=11<br>- scRNA-seq: female n=10, male n=9<br>- Spatial transcriptomics: female n=0, male n=2<br>- Peripheral blood plasma: female n=24; male n=16
Reporting on race, ethnicity, or other socially relevant groupings	Race/ethnicity were were not included in the study design. No exclusions were made based on race, ethnicity, sex, gender, age or social factors.<br><br>- Race: Caucasian (n=74)<br>- Country of birth: German (n=18), Bulgaria (n=1), Serbia (n=1), Poland (n=1), Turkey (n=1), Russia (n=1)
Population characteristics	Patient characteristics necessary for clinical interpretation are documented in Supplementary Table 1.
Recruitment	All patient samples were collected from adult patients after obtaining written informed consent in accordance with the Declaration of Helsinki, including consent for publication of de-identified data derived from these samples. No participant compensation was provided. No statistical methods were used to pre-determine sample sizes due to the exploratory nature of the study, but our sample sizes are similar to those reported in previous publications.<br>Samples were collected between 08/20 to 07/21 (lymph node samples for spatial and scRNA-seq) and 03/21 to 02/24 (plasma samples for ELISA) and all samples collected in this timeframe were used in this study, thus eliminating concerns of self-selection bias.
Ethics oversight	The Ethics Committee of the Medical Faculty of the University of Heidelberg approved the study (S-057/2019).

Note that full information on the approval of the study protocol must also be provided in the manuscript.

Field-specific reporting

Please select the one below that is the best fit for your research. If you are not sure, read the appropriate sections before making your selection.

☒ Life sciences ☐ Behavioural & social sciences ☐ Ecological, evolutionary & environmental sciences

For a reference copy of the document with all sections, see nature.com/documents/nr-reporting-summary-flat.pdf

Life sciences study design

All studies must disclose on these points even when the disclosure is negative.

Sample size	Given the exploratory nature of our project, the scarcity of human lymph node samples and inherent variability across patient samples, no statistical methods were used to pre-determine sample sizes, but our sample sizes are similar to those reported in previous publications. In this study we conducted single-cell RNA sequencing (scRNA-seq) across a cohort of 19 patient samples aiming for a maximum 2,000 cells per population per sample. Spatial transcriptomics data was collected in 2 representative samples as selected by board certified pathologists.

Data exclusions	In scRNA-seq cells with < 500 detected genes and >25% mitochondrial counts were discarded. Such quality control steps are customary in the field and were set after inspection of the data, and not pre-established. In spatial transcriptomics dataset cells with > 50 transcripts per cell were retained for further analysis. In the other datasets presented in this paper no data was excluded.
Replication	For experiments using patient-derived primary material biological replicates were used as indicated in the figure legends. Because of the limited availability of patient-derived primary material, full replication of all experiments in independent cohorts was not feasible. To support reproducibility under these constraints, we implemented standardized protocols, included internal technical replicates where possible, and confirmed key results across independent donors when material was available. We did not observe any failed replication attempts for the experiments that could be repeated, but we acknowledge that some findings are based on single experimental series and should be interpreted with caution. Validation in large bulk transcriptomics cohorts was used to substantiate statements about disease entities.
Randomization	Not applicable (no treatment groups).
Blinding	This study was performed unblinded due to logistical constraints, i.e. blinding was not feasible because sample handling and allocation were inherently linked to the clinical workflow or the clinician who enrolled patients also processed and annotated the specimens. However, outcome measures were based on predefined, quantitative readouts, limiting the risk of observer bias.

Reporting for specific materials, systems and methods

We require information from authors about some types of materials, experimental systems and methods used in many studies. Here, indicate whether each material, system or method listed is relevant to your study. If you are not sure if a list item applies to your research, read the appropriate section before selecting a response.

Materials & experimental systems

n/a	Involved in the study	
☐	☒	Antibodies
☒	☐	Eukaryotic cell lines
☒	☐	Palaeontology and archaeology
☒	☐	Animals and other organisms
☒	☐	Clinical data
☒	☐	Dual use research of concern
☒	☐	Plants

Methods

n/a	Involved in the study	
☒	☐	ChIP-seq
☐	☒	Flow cytometry
☒	☐	MRI-based neuroimaging

Antibodies

Antibodies used	Antibody information is documented in Supplementary Table 9.
Validation	Antibodies were validated by the commercial supplier and/or by titration in flow cytometry.

Plants

Seed stocks	n/a
Novel plant genotypes	n/a
Authentication	n/a

Flow Cytometry

Plots

Confirm that:

☒ The axis labels state the marker and fluorochrome used (e.g. CD4-FITC).

☒ The axis scales are clearly visible. Include numbers along axes only for bottom left plot of group (a 'group' is an analysis of identical markers).

☒ All plots are contour plots with outliers or pseudocolor plots.

☒ A numerical value for number of cells or percentage (with statistics) is provided.

Methodology

Sample preparation	Lymph nodes were processed, frozen, and thawed using the methods outlined in the Methods section.
Instrument	FACSAria Fusion (BD Biosciences), Cytek Aurora Spectral Flow Cytometer (Cytek Biosciences)
Software	FlowJo
Cell population abundance	Variable across samples, sorted cell numbers per sample can provided upon request.
Gating strategy	The gating strategy for LN stromal cells is outlined in Extended Data Fig. 1.

☒ Tick this box to confirm that a figure exemplifying the gating strategy is provided in the Supplementary Information.
